# Conservation Genomics of Two Threatened Subspecies of Northern Giraffe: The West African and the Kordofan Giraffe

**DOI:** 10.3390/genes13020221

**Published:** 2022-01-25

**Authors:** Raphael T. F. Coimbra, Sven Winter, Barbara Mitchell, Julian Fennessy, Axel Janke

**Affiliations:** 1 Senckenberg Biodiversity and Climate Research Centre, 60325 Frankfurt am Main, Germany; sven.winter@senckenberg.de (S.W.); barmit96@yahoo.de (B.M.); 2 Institute for Ecology, Evolution and Diversity, Goethe University, 60439 Frankfurt am Main, Germany; 3 Giraffe Conservation Foundation, Windhoek 9000, Namibia; julian@giraffeconservation.org; 4 LOEWE Centre for Translational Biodiversity Genomics, 60325 Frankfurt am Main, Germany

**Keywords:** conservation, genomic diversity, inbreeding, northern giraffe, population genomics, population structure, runs of homozygosity, stairway plot

## Abstract

Three of the four species of giraffe are threatened, particularly the northern giraffe (*Giraffa camelopardalis*), which collectively have the smallest known wild population estimates. Among the three subspecies of the northern giraffe, the West African giraffe (*Giraffa camelopardalis peralta*) had declined to 49 individuals by 1996 and only recovered due to conservation efforts undertaken in the past 25 years, while the Kordofan giraffe (*Giraffa camelopardalis antiquorum*) remains at <2300 individuals distributed in small, isolated populations over a large geographical range in Central Africa. These combined factors could lead to genetically depauperated populations. We analyzed 119 mitochondrial sequences and 26 whole genomes of northern giraffe individuals to investigate their population structure and assess the recent demographic history and current genomic diversity of West African and Kordofan giraffe. Phylogenetic and population structure analyses separate the three subspecies of northern giraffe and suggest genetic differentiation between populations from eastern and western areas of the Kordofan giraffe’s range. Both West African and Kordofan giraffe show a gradual decline in effective population size over the last 10 ka and have moderate genome-wide heterozygosity compared to other giraffe species. Recent inbreeding levels are higher in the West African giraffe and in Kordofan giraffe from Garamba National Park, Democratic Republic of Congo. Although numbers for both West African and some populations of Kordofan giraffe have increased in recent years, the threat of habitat loss, climate change impacts, and illegal hunting persists. Thus, future conservation actions should consider close genetic monitoring of populations to detect and, where practical, counteract negative trends that might develop.

## 1. Introduction

Since the 1980s, giraffe (*Giraffa* spp.) numbers in the wild have declined by ~30% [1]. Although giraffe are considered a single species and are listed as “Vulnerable” by the International Union for Conservation of Nature (IUCN) [2], the latest genetic assessments show that giraffe more likely consist of either three [3,4] or four species [5,6,7]. Among those, and occurring across northern sub-Saharan Africa, is the northern giraffe (*Giraffa camelopardalis* sensu Fennessy et al. [5]), the least populous giraffe species with <5920 individuals remaining in the wild [1]. The four species taxonomy originally proposed by Fennessy et al. [5] and adopted herein includes three genetically recognized extant subspecies of the northern giraffe: the West African (*Giraffa camelopardalis peralta*), the Kordofan (*Giraffa camelopardalis antiquorum*), and the Nubian giraffe (*Giraffa camelopardalis camelopardalis* senior synonym of *Giraffa camelopardalis rothschildi*).

The West African giraffe was widely distributed throughout West and North Africa during Paleolithic times, including the Mediterranean coastline [8]. At the end of the 19th century, it was still present in the Sudano-Sahelian zone from Senegal to Nigeria [9]. However, illegal hunting, habitat loss, and fragmentation extirpated all populations outside Niger, and by 1996 only 49 individuals remained in the “Giraffe Zone”, an area southeast of Niger’s capital Niamey [8,9,10]. Since then, concerted conservation efforts by the Government of Niger and conservation partners have helped West African giraffe numbers to increase to >600 individuals [1,10,11]. As a result of this successful population recovery, the West African giraffe was downlisted from “Endangered” to “Vulnerable” on the IUCN Red List in 2018 [11]. In addition, in November 2018, eight West African giraffe individuals were translocated to the Gadabedji Biosphere Reserve in eastern Niger to establish an independent satellite population back in its natural range—as part of a collaboration between the Government of Niger and the Giraffe Conservation Foundation (GCF) [12].

The Kordofan giraffe was formerly widespread across savanna woodlands and the Sahel in Central Africa [13], with its numbers estimated at >13,500 during the 1980s [14]. Today, small populations totaling <2300 individuals are scattered in isolated patches across Cameroon, Central African Republic, Chad, Democratic Republic of the Congo (DRC), and South Sudan, mostly inside protected areas [1]. This drastic decline of >80% over the last 35 years has been driven by illegal hunting, habitat loss, and civil unrest, resulting in the IUCN Red Listing of the Kordofan giraffe as “Critically Endangered” [14]. In the last decade, concerted conservation programs for Kordofan giraffe by GCF, the African Parks Network (APN), various governments, and other conservation partners in key areas such as DRC’s Garamba National Park (NP) and Chad’s Zakouma NP, has fortunately facilitated populations to slowly rebound [1,14].

Small and isolated wildlife populations are particularly vulnerable to loss of genetic diversity and increased inbreeding [15]. As such, conservation genetics and the analyses of DNA data can assist with effective conservation planning and management as they enable population stratification surveys and the estimation of relevant genetic and demographic parameters for monitoring [16,17]. In giraffe, previous studies that included samples of northern giraffe subspecies focused on phylogeography and speciation [3,4,5,6,18,19,20]. To date, nuclear genetic diversity was only examined at 14 microsatellite loci for West African and Nubian giraffe [21], and more recently, at single nucleotide polymorphism (SNP) sites for a few individuals in a comparison between different giraffe species [7]. However, neither of these latter studies focused on conservation genetics aspects of northern giraffe subspecies.

Here, our goal was to investigate patterns of population structure within the northern giraffe and perform a detailed assessment of recent population size changes and genomic diversity (i.e., heterozygosity and inbreeding) in West African and Kordofan giraffe to aid the development of conservation management strategies. The high resolution provided by whole-genome data and an improved sampling over Coimbra et al. [7] enabled these in-depth analyses. Of particular interest was to investigate if the recent population bottleneck underwent by the West African giraffe is detectable from their genomes. In addition, this is the first genomic study of the Kordofan giraffe in Zakouma NP, Chad. Despite increasing numbers of West African giraffe in Niger and Kordofan giraffe in Zakouma NP, these populations are still threatened and need targeted ongoing management and protection. Thus, their genetic characterization is critical as they represent potential source populations for future translocations across their respective former ranges.

## 2. Materials and Methods

### 2.1. Sampling and DNA Extraction

Skin biopsy samples from 20 West African giraffe from the Kouré plateau (“Giraffe Zone”), Niger, and nine Kordofan giraffe from Zakouma NP, Chad, were collected by the GCF and partners using remote biopsy darting and preserved in 80% ethanol. Sampling was conducted with country-specific research permits following ethical guidelines of the respective governments. DNA was isolated using either a NucleoSpin Tissue kit (Macherey-Nagel) or a standard phenol-chloroform protocol [22].

### 2.2. PCR Amplification, Sequencing, and Alignment of Mitochondrial Loci

We amplified the complete cytochrome b (*Cytb*) gene and a partial mitochondrial control region (CR) via polymerase chain reaction (PCR) using giraffe-specific primers (*Cytb*: 5′ TGAAAAACCATCGTTGTCGT 3′ and 5′ TGGGAGTATATTAATAGC 3′; CR: 5′ TACACTGGTCTTGTAAGC 3′ and 5′ TCGCTTTGGTGTTTAAGC 3′). Amplification was performed in a final volume of 15 μL containing ~10 ng of DNA, 7.5 μL of Taq DNA Polymerase 2X Master Mix with 2.0 mM MgCl_2_ (VWR International), 0.35 μL of 10 pmol/μL of each primer and the remaining volume of desalted water. Thermal cycling consisted of an initiation step at 95 °C for 5 min, followed by 35 cycles of denaturation at 95 °C for 30 s, annealing at 50 °C for 30 s, and elongation 72 °C for 1 min, with a final elongation step at 72 °C for 5 min. PCR products were examined by electrophoresis on a 1% agarose gel with ethidium bromide. Amplicons were sequenced with the BigDye Terminator v3.1 cycle sequencing kit (Applied Biosystems). In some cases, an additional primer (5′ TCGGCACAAATCTAGTCG 3′) was used for sequencing the second half of the *Cytb*. Sequencing products were analyzed on an ABI 3730 DNA Analyzer (Applied Biosystems) and chromatograms were inspected in Geneious Prime v2020.1.2 (https://www.geneious.com/ (accessed on 14 July 2020)).

**Figure 1:**
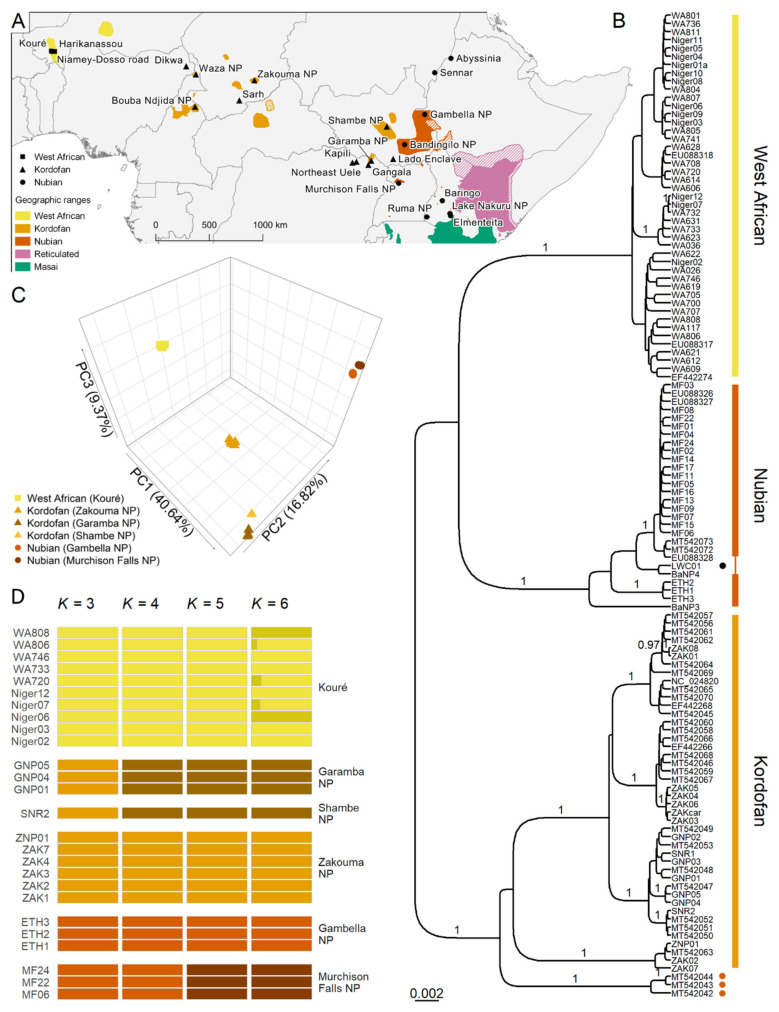
Population structure in the northern giraffe. (**A**) Geographical distribution of northern giraffe subspecies (colored shadings) in sub-Saharan Africa and sampling locations. Hatched areas indicate possible but unconfirmed range of northern giraffe populations. (**B**) Bayesian phylogenetic tree of northern giraffe subspecies based on mitochondrial control region and *Cytb* sequences. The complete tree with 356 sequences of all giraffe species is shown in Supplementary Figure S1. Posterior probability (PP) support is denoted for branches with PP ≥ 0.95. Tips marked with a dark orange circle denote museum specimens from Abyssinia (present-day Ethiopia) and Sennar, Sudan. The tip marked with a black circle is a potential hybrid between Nubian and reticulated giraffe. (**C**) PCA of 192,177 unlinked SNPs from 26 northern giraffe individuals. PC1 separates the West African giraffe from other subspecies, PC2 separates Kordofan and Nubian giraffe, and PC3 distinguishes Kordofan giraffe individuals from different conservation areas. Shapes represent subspecies and colors indicate sampling location. (**D**) Admixture analysis based on the same SNP dataset assuming *K* from 3 to 6. Clusters observed at *K* = 3 reflect the three northern giraffe subspecies. From *K* = 4 to 5, populations of Kordofan and Nubian giraffe from different national parks form separate clusters. No further biologically meaningful clusters are observed at *K* = 6. Colors indicate an individual’s cluster membership. Run likelihoods per *K* are shown in Supplementary Figure S2.

The sampled individuals were added to a mitochondrial dataset containing 327 wild giraffe sequences publicly available at GenBank [4,5,6,7,18,19,20,21,23,24,25], representing all species and subspecies. Sample identifiers, locality, and accession numbers for all sequences analyzed here are shown in Figure 1A and Supplementary Table S1. Multiple sequence alignments were built with the E-INS-i algorithm in MAFFT v7.475 [26], including all 356 wild giraffe sequences and the okapi (*Okapia johnstoni*) as an outgroup.

### 2.3. Phylogenetic Inference on Mitochondrial Data

Phylogenetic inference on mitochondrial CR and *Cytb* sequences—including 45 West African, 44 Kordofan, and 30 Nubian giraffe—was performed in BEAST v2.6.4 [27]. Site and clock models were estimated separately for each partition, whereas tree inference was linked. The transition/transversion split option for substitution models in bModelTest v1.2.1 [28] was used in combination with a strict clock and coalescent exponential population tree prior. Three independent Markov chain Monte Carlo (MCMC) were run for 75 million generations sampling every 7500th. Trace files were analyzed in Tracer v1.7.1 [29] to ensure chain convergence and appropriate effective sample sizes (ESS). Tree log files from all runs were combined in LogCombiner with a 50% burn-in, and a maximum clade credibility (MCC) tree was summarized in TreeAnnotator with a 33% burn-in. Both LogCombiner and TreeAnnotator are part of the BEAST2 package. The MCC tree was visualized with ggtree v1.16.1 [30].

### 2.4. Whole-Genome Re-Sequencing and Read Mapping

To perform population structure and genomic diversity analyses, we re-sequenced whole genomes of five West African and five Kordofan giraffe individuals from the newly obtained samples. Libraries were prepared and sequenced at Novogene on an Illumina NovaSeq 6000 (2 × 150 bp, 350 bp insert size). Short reads of five West African, five Kordofan, and six Nubian giraffe from Fennessy et al. [5] and Coimbra et al. [7], and an okapi from Agaba et al. [31] were added to the dataset generated here. For details on the dataset, see Figure 1A and Supplementary Table S2.

Read quality control and trimming were performed using fastp v0.20.0 [32] with base correction and low complexity filter enabled. Adapters and polyG stretches in read tails were automatically detected and removed. A sliding window of 4 bp, moving from the 3′ to the 5′ end, dropped the bases in the window if the mean base quality was <15. Reads were discarded if they were shorter than 36 bp, contained >40% of bases with quality <15, or included more than five undetermined bases. The remaining read pairs were mapped to a chromosome-scale Masai giraffe s. str. (*Giraffa tippelskirchi tippelskirchi*) genome assembly (GCA_013496395) [33] with BWA-MEM v0.7.17 (r1188) [34] and sorted with samtools v1.10 [35]. Lane level BAMs were merged per sample with samtools prior to deduplication of read alignments with MarkDuplicates from Picard v2.21.7 (http://broadinstitute.github.io/picard/ (accessed on 21 January 2020)). Realignment around indels was performed with GATK v3.8.1 [36]. Reads flagged as unmapped, secondary/supplementary alignment, failed quality checks, or PCR/optical duplicates were removed from the BAM files with samtools while keeping only reads mapped in a proper pair. Reads mapped to regions identified as known Cetartiodactyla repeat elements by RepeatMasker v4.0.7-open (http://repeatmasker.org (accessed on 1 March 2018)) or to scaffolds assigned as sex chromosomes were also excluded.

### 2.5. SNP Calling and Linkage Pruning

SNPs were called with ANGSD v0.933 [37] using samtools’ model for genotype likelihood estimation. Base alignment quality (BAQ) computation [38] and mapping quality adjustment (flag -C 50) were enabled. A minimum score of 30 was set for both mapping and base qualities. The minimum and maximum depth thresholds were set to *d* ± (5 × *MAD*), where *d* is the median of the global site depth distribution and *MAD* is the median absolute deviation. Sites with a *p*-value < 1 × 10^−6^ for strand bias, heterozygous bias, or Hardy-Weinberg equilibrium (HWE) tests were discarded. Only biallelic SNPs called with a *p*-value < 1 × 10^−6^ in at least 90% of the individuals, with a minor allele frequency (MAF) ≥ 0.05, were retained.

SNPs called by ANGSD were pruned for linkage disequilibrium (LD) with ngsLD v1.1.1 [39]. LD was estimated as *r*^2^ values for all SNP pairs up to 500 kbp apart. An LD decay curve was plotted for a random sample of 0.05% of all estimated *r*^2^ values, with a bin size of 250, to determine appropriate thresholds for linkage pruning. Sites were pruned assuming a maximum distance of 75 kbp between SNPs and *r*^2^ ≥ 0.1.

### 2.6. Population Structure Analyses

Genotype likelihoods of LD-pruned SNPs were used to calculate a covariance matrix in PCAngsd v1.01 [40], which was then loaded into R v3.6.2 [41] to perform a principal component analysis (PCA) using the prcomp() function. Individual ancestries were inferred in NGSadmix v32 [42] for a range of numbers (one to six) of ancestry components (*K*), each with 100 replicates. The replicate with the highest likelihood for each *K* ≥ 3 was shown as an admixture plot and run likelihoods per *K* were shown as a boxplot.

### 2.7. Ancestral Demography

We inferred the ancestral demography of West African and Kordofan giraffe to assess recent changes in their effective population sizes (*N_e_*) based on the unfolded site frequency spectrum (SFS). The Nubian giraffe was not included due to sample size limitation. To polarize SNPs during the SFS estimation, we generated a genome consensus sequence for the okapi using ANGSD (flag -doFasta 1). BAQ computation and mapping quality adjustment were enabled. Sites with mapping or base qualities <30, minimum depth <4, or maximum depth above the 95th percentile of the sample’s depth distribution were discarded. We then estimated the site allele frequencies for the referred giraffe subspecies in ANGSD (flag -doSaf 1) using the okapi consensus sequence as ancestral. Quality filters were set as described for SNP calling; however, filters for HWE, minimum MAF, and SNP *p*-value were removed to avoid distorting the SFS [43]. ANGSD’s companion program realSFS was used to convert the site allele frequencies into the unfolded SFS. Demographic histories were inferred from the unfolded SFS with Stairway Plot v2.1.1 [44] using recommended default settings. Results were scaled by a mutation rate of 2.12 × 10^−8^ substitutions per site per generation estimated for the giraffe [45] and a generation time of 10 years [2].

### 2.8. Nuclear Genomic Diversity

To investigate the nuclear genomic diversity of the northern giraffe subspecies, we measured genome-wide heterozygosity and inbreeding levels among the 26 re-sequenced individuals.

Genome-wide heterozygosity was estimated based on the folded SFS. Site allele frequencies were estimated per sample in ANGSD (flag -doSaf 1) using the reference genome as ancestral. BAQ computation and mapping quality adjustment were enabled. A minimum score of 30 was set for both mapping and base qualities, and a maximum depth cut-off was set to the 95th percentile of the sample’s depth distribution. The per sample folded SFSs were generated in realSFS (flag -fold 1) with 200 bootstrap replicates. Heterozygosity was then calculated in R as the percentage of heterozygous sites.

Levels of genomic inbreeding were assessed by analyzing segments of homozygosity-by-descent (HBD) among individuals. SNPs were called in ANGSD as described previously; however, no MAF filtering or LD pruning were performed. To generate a VCF file for further analysis, genotypes were called in bcftools v1.14 (https://samtools.github.io/bcftools/ (accessed on 22 October 2021)) with the mpileup and call pipeline at SNP sites identified by ANGSD (flag -T). Bcftools filter and view were used to convert genotypes with GQ < 20 to missing data and keep only biallelic SNPs with data for at least 90% of the individuals, QUAL ≥ 30, MQ ≥ 30, and within the depth thresholds set in ANGSD. RZooRoH v0.3.0 [46] was used to identify HBD segments of multiple age/length-related classes and estimate the realized inbreeding coefficients (F_HBD_) from genotype probabilities. F_HBD_ levels represent the proportion of the autosome genome contained in HBD segments of different classes. Longer HBD segments correspond to more recent inbreeding, while shorter HBD segments correspond to more ancestral inbreeding. We set a hidden Markov model (HMM) with 15 HBD classes with predefined rates following an exponential series of base two (2*^n^*, where *n* = 1 to 15) and a non-HBD class. The rate of an HBD class is associated to the expected length of the HBD segments in that class, and thus it is also informative of the age of past inbreeding events. An HBD class *k* of rate *R_k_* corresponds to ancestors inbreeding approximately 0.5 × *R_k_* generations ago. HBD classes with *R_k_* ≥ 1024 are more likely to reflect ancestral *N_e_* than inbreeding.

## 3. Results

Sequencing of mitochondrial markers was largely successful and only *Cytb* could not be sequenced for individuals Niger02 and ZAK02. A Bayesian mitochondrial phylogeny based on *Cytb* (1140 bp) and CR (418 bp) alignments for the three northern giraffe subspecies is shown in Figure 1B. A complete tree with 356 giraffe sequences representing all subspecies is shown in Supplementary Figure S1. The relationships among mitochondrial lineages of giraffe generally conform with previous analysis [4,7].

The new samples of West African and Kordofan giraffe sequenced here grouped within their respective subspecies as expected. The West African giraffe is more closely related to the Nubian giraffe, and the clade West African plus Nubian is sister to the Kordofan giraffe. However, in both cases, the branch posterior probability (PP) is low (PP = 0.79). Within Kordofan giraffe, we find lineages with full PP support that are geographically structured (western—Zakouma NP, Chad; Zakouma NP + Sarh, Chad; Zakouma NP, Chad + Waza NP and Bouba Ndjida NP, Cameroon + Dikwa, Nigeria; eastern—DRC + Sudan), although the relationships between them are not entirely resolved.

Three museum samples of Nubian giraffe from Sennar, Sudan, and Abyssinia (present-day Ethiopia) sequenced by Petzold et al. [4] form a monophyletic lineage that also groups with Kordofan giraffe. Another two museum samples from Bakel, Senegal, form a clade closely related to the reticulated giraffe (*Giraffa reticulata*), albeit with low posterior probability (PP = 0.74; Supplementary Figure S1). The reticulated giraffe individual LWC01 groups with the Nubian giraffe and is likely a hybrid, as suggested previously [6].

A PCA of 192,177 unlinked SNPs from 26 northern giraffe individuals shows four distinct genetic clusters (Figure 1C). PC1 separates the West African giraffe from other subspecies, PC2 separates Kordofan and Nubian giraffe, and PC3 distinguishes Kordofan giraffe individuals from different conservation areas. Analyses of individual ancestries based on the same dataset show an identical pattern of population structure to that found in the PCA (Figure 1D). At *K* = 3, the observed clusters reflect the three northern giraffe subspecies. At *K* = 4, Kordofan giraffe individuals from Garamba NP in the DRC and Shambe NP in South Sudan cluster separately from those from Zakouma NP in Chad. Lastly, at *K* = 5, Nubian giraffe individuals from Gambella NP in Ethiopia and Murchison Falls NP in Uganda form different groups. No further biologically meaningful clusters are detected at *K* = 6. Run likelihoods per *K* are shown in Supplementary Figure S2.

**Figure 2:**
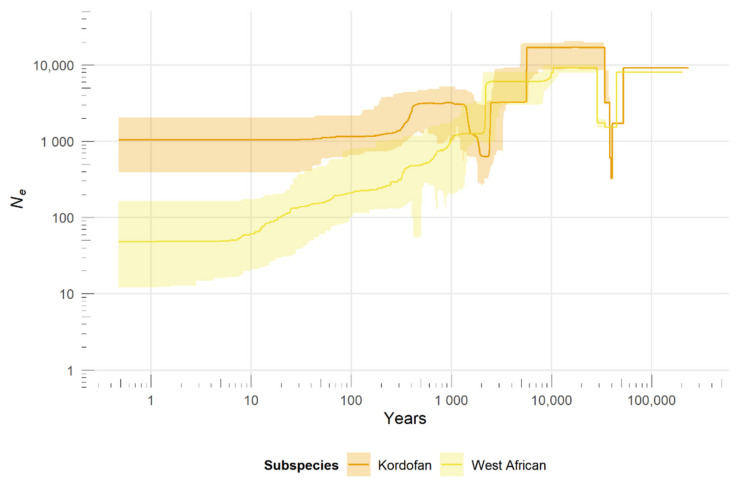
Demographic history of West African and Kordofan giraffe. Recent changes in *N_e_* over time were inferred based on the unfolded site frequency spectrum (SFS) using the stairway plot method. Axes were scaled by a mutation rate of 2.12 × 10^−8^ substitutions per site per generation and a generation time of 10 years. Colors represent subspecies. Solid lines indicate the median estimates of *N_e_* and shaded areas correspond to the 95% confidence intervals.

The reconstruction of population size changes over the recent past (Figure 2) shows a gradual decline in West African giraffe numbers in the last 10 ka, with median *N_e_* dropping from ~8000 to currently ~50. The Kordofan giraffe went through a population decline ~5.5 ka ago, followed by a rebound ~2 ka ago and another decline between ~40–400 years ago. In the last 10 ka, the Kordofan giraffe’s median *N_e_* decreased from ~17,000 to ~1000. Both subspecies show a bottleneck between ~29–51 ka ago; however, that is nearing the ancestral end of the stairway plot’s estimates and thus cannot be interpreted reliably.

**Figure 3:**
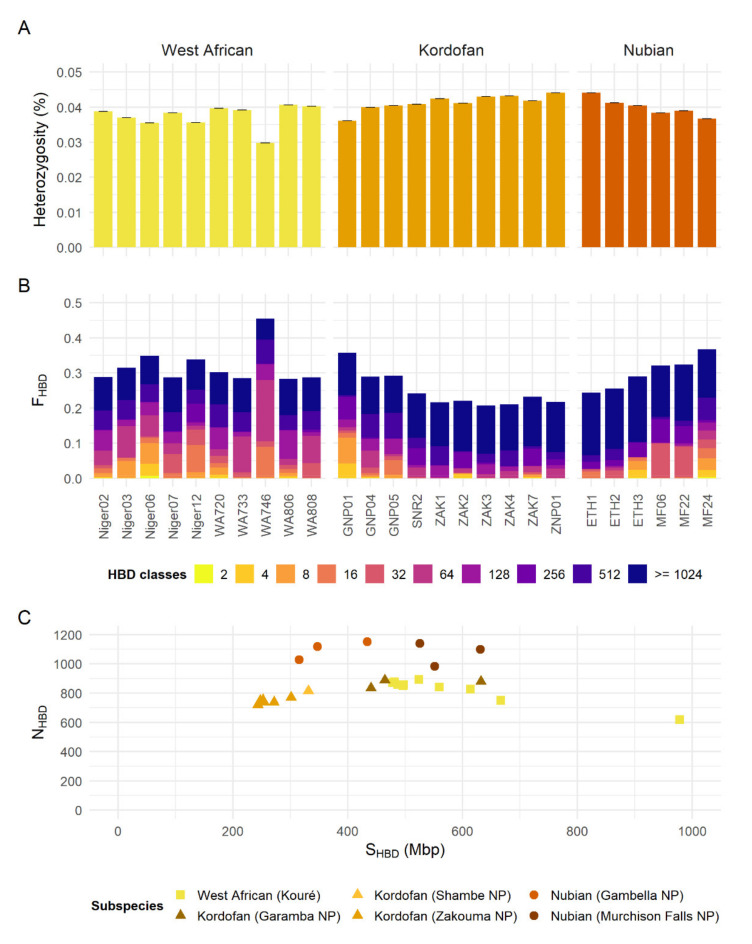
Genomic diversity among the northern giraffe subspecies. (**A**) Genome-wide heterozygosity estimates per individual for each subspecies. Heterozygosity was measured as an individual’s percentage of heterozygous sites. Whiskers on top of each bar represent the standard deviation. (**B**) Realized inbreeding coefficients (F_HBD_) per individual for each subspecies. The proportion of the genome assigned to each of 15 age/length-related classes of homozygosity-by-descent (HBD) is shown in different colors. HBD classes with rates equal to {2, 4, 8, 16, …, 32,768} correspond to ancestors inbreeding approximately 0.5 × *R_k_* generations ago, where *R_k_* is the rate of the class *k*. HBD classes with *R_k_* ≥ 1024 are more likely to reflect *N_e_* in the distant past than inbreeding, and thus were clumped in the plot. See also Supplementary Figure S3. (**C**) Number (N_HBD_) versus total length (S_HBD_) of HBD segments per individual for each subspecies. Note that HBD segments <100 kbp were removed. Shapes represent subspecies and colors indicate sampling location.

Genome-wide heterozygosity estimates show a median around 0.038% (0.030–0.041%) for the West African giraffe, 0.042% (0.036–0.044%) for the Kordofan giraffe, and 0.040% (0.037–0.044%) for the Nubian giraffe (Figure 3A). F_HBD_ levels obtained after excluding HBD classes with *R_k_* ≥ 1024 (F_HBD<1024_) correspond to a median of 0.202 (0.180–0.395) for the West African giraffe, 0.091 (0.070–0.236) for the Kordofan giraffe, and 0.134 (0.066–0.229) for the Nubian giraffe (Figure 3B). Regarding Kordofan giraffe populations, F_HBD<1024_ levels are higher in individuals from Garamba NP (0.183–0.236) than in those from Shambe NP (0.115) and Zakouma NP (0.070–0.091). For Nubian giraffe populations, F_HBD<1024_ levels are higher in individuals from Murchison Falls NP (0.164–0.229) than in those from Gambella NP (0.066–0.103). The number and total length of HBD segments among individuals of different subspecies and populations reflect the pattern observed for F_HBD_ (Figure 3C). HBD classes associated with more recent inbreeding (*R_k_* ≤ 128) were generally more abundant in West African giraffe individuals (Figure 3B and Supplementary Figure S3).

## 4. Discussion

The three subspecies of northern giraffe are widely distributed over a large geographic range in northern sub-Saharan Africa. Congruent with previous results using limited sampling [7], northern giraffe are split into three distinct clades with little to no genetic exchange between them. This pattern has been previously observed in the analyses of mitochondrial DNA [4,6,7]. Moreover, northern giraffe subspecies have relatively low genome-wide heterozygosity and moderate levels of F_HBD_ when compared to mean estimates among other 78 mammalian species (heterozygosity = 0.273% ± 0.223%, F_HBD_ = 0.075 ± 0.134) [47]. However, considering their current small population sizes, their genomic diversity is relatively high compared to median estimates for other giraffe subspecies with much larger populations, i.e., South African (*Giraffa giraffa giraffa*; heterozygosity = 0.018% and F_HBD<1280_ = 0.174), Angolan (*Giraffa giraffa angolensis*; heterozygosity = 0.016% and F_HBD<1280_ = 0.242), and Masai giraffe s. str. (heterozygosity = 0.024% and F_HBD<1280_ = 0.246) [7].

Genomic data show that the West African giraffe underwent a steady *N_e_* decline over the last 10 ka, consistent with the contraction of its historical geographical range [8]. However, despite the known records of a drastic reduction in its census population size during the last century [11], no concurrent accentuated *N_e_* bottleneck was detected during that period. Furthermore, median *N_e_* values estimated for the present are within a plausible range; an order of magnitude smaller than the current estimated census population [48]. These findings complement a previous demographic reconstruction for the subspecies [7] and provide a more complete picture of the population history of the West African giraffe.

The West African giraffe has recently recovered from only 49 individuals to over 600 in the last three decades, all living in a restricted area in Niger. This explains the absence of any population structure. The generally lower median heterozygosity and higher F_HBD<1024_ observed for West African giraffe relative to other northern giraffe subspecies is consistent with its recent population history. However, its genomic diversity is not as alarming as expected for a population that is only recently recovering from a sharp decline, especially when compared to southern and Masai giraffe [7]. This is encouraging and demonstrates that the conservation efforts undertaken in Niger were timely to prevent the deleterious effects of inbreeding depression. The remaining genomic diversity in the West African giraffe should aid the survival of the individuals translocated to the Gadabedji Biosphere Reserve and lessens the concerns regarding the choice of suitable individuals. However, genetic monitoring of this recently established satellite population is recommended to monitor potential inbreeding at early stages and if needed, advise further augmentation.

The Kordofan giraffe shows no mitochondrial haplotype sharing between samples from the eastern (DRC and South Sudan) and western (Chad, Cameroon, and Nigeria) populations across its range. A similar spatial structure is also observed in its nuclear genome and is likely attributed to isolation-by-distance due to the large geographical distance separating the remaining small, isolated populations across East-Central Africa. Given the observed genetic differences, it is advisable to manage the eastern and western populations of Kordofan giraffe separately, unless in time there are no other viable options. Furthermore, the Kordofan giraffe’s *N_e_* estimated at the present is lower, but in line with, recent estimates of census population size [1,14]. However, this finding should be interpreted with caution as the presence of population structure may confound the outcome of demographic inferences [49]. A larger sample size per population would be required for more accurate reconstructions of recent population size changes within the Kordofan giraffe.

The Kordofan giraffe’s higher overall heterozygosity and lower F_HBD<1024_ compared to the West African giraffe conform to its relatively larger *N_e_*. When populations are analyzed independently, individuals from Garamba NP are more inbred than those from Shambe NP and Zakouma NP, as shown by their higher F_HBD<1024_, and higher number and longer total length of HBD segments. The Kordofan giraffe of Garamba NP and adjacent hunting reserves is the last remaining population in the DRC. It has decreased from ~400 individuals in the early-1990s to a low of 22, before rebounding in the past decade to a current estimate of 65 individuals [50,51]. Although recent conservation and management actions by the APN and partners have improved the conservation status of the Garamba NP population, genetic monitoring and population augmentation should be considered to minimize the chances of future inbreeding depression. Conversely, the population from Zakouma NP represents the largest population of Kordofan giraffe with over 60% of the total wild population [1,14]. With an increasing population trend over the past decade and low levels of recent inbreeding, the conservation status of giraffe in Zakouma NP is positive under the improved private-public management.

Fennessy et al. [5] found that the Rothschild’s giraffe (*G. c. rothschildi*) from Kenya and Uganda, and the Nubian giraffe (*G. c. camelopardalis*) from Ethiopia and South Sudan are genetically indistinguishable, and thus should be subsumed under the latter name. Petzold et al. [4] argue that what Fennessy et al. [5] consider Nubian giraffe should instead be named Rothschild’s giraffe, and that the original Nubian giraffe occurred further north, in Sudan and northern Ethiopia, and today are likely extinct. This finding was based on the analysis of mitochondrial sequences, including three 18th century museum specimens of Nubian giraffe from its type locality (Sennar, Sudan, and Abyssinia). They found that these three individuals formed a monophyletic clade more closely related to the Kordofan giraffe. Thus, Petzold et al. [4] interpreted that Rothschild’s and Nubian giraffe should be considered two separate subspecies, with Rothschild’s more closely related to West African giraffe, and Nubian more closely related to Kordofan giraffe. We observed the same patterns in our phylogenetic analysis. However, based solely on mitochondrial data, it is not possible to discard the hypothesis that these individuals represent an ancestral lineage of Kordofan giraffe. A more detailed investigation of the taxonomic status of those individuals is warranted, if possible, using modern nuclear genome sequencing techniques appropriate for museum specimens [52]. Until then, northern giraffe occurring in Kenya, Uganda, Ethiopia, and South Sudan should remain as Nubian giraffe.

Our study provides an important contribution to the understanding of the demographic histories and spatial patterns of genetic diversity in West African and Kordofan giraffe. However, due to the difficulty of obtaining samples, especially in areas of civil and political unrest, the sample sizes per population were limited and some populations are missing from genomic analysis, particularly for the Kordofan giraffe. Therefore, future studies with a denser sampling across more populations are warranted to form a more complete picture of the geographic and genetic configuration of Kordofan giraffe populations and potentially assess gene flow between northern giraffe subspecies.

## 5. Conclusions

The West African and Kordofan giraffe have retained a moderate genomic diversity despite their recent declines in numbers, and the small and fragmented distribution of their populations. However, despite their numbers increasing in some populations, the continued threats of habitat loss, climate change, and poaching should not be ignored. Targeted conservation genetic monitoring is recommended to assess and, where appropriate, effectively counteract potential negative trends that might develop. Long-term conservation of the northern giraffe is critical for maintaining the biodiversity of the world’s tallest mammal.

## Data Availability

Raw sequencing reads generated during this study are available at NCBI Short Read Archive under the BioProject accession PRJNA772549. Mitochondrial nucleotide sequences generated during this study are available at GenBank under the accession numbers OK558543–OK558598.

## Supplementary Materials

The following supporting information can be downloaded at: https://www.mdpi.com/article/10.3390/genes13020221/s1, Figure S1: Bayesian mitochondrial phylogeny of giraffe species and subspecies; Figure S2: Run likelihoods per K; Figure S3: Proportion of the genome associated with different HBD classes; Table S1: Sample details for mitochondrial dataset; Table S2: Sample details and mapping statistics for re-sequenced individuals.
