# Supplementary material for: Conservation Genomics of Two Threatened Subspecies of Northern Giraffe: The West African and the Kordofan Giraffe
Source: Genes (Basel). 2022 Jan 25;13(2):221. doi: 10.3390/genes13020221 (PMC8872558; doi:10.3390/genes13020221)
Supplement: Supplementary file 1 [file genes-13-00221-s001.zip › supplementary_materials.pdf]

Supplementary Materials

Conservation Genomics of Two Threatened Subspecies of Northern Giraffe: The West African and the Kordofan Giraffe

Raphael T. F. Coimbra [1,2,*], Sven Winter [1], Barbara Mitchell [1,2], Julian Fennessy [3], and Axel Janke [1,2,4,*]

[1] Senckenberg Biodiversity and Climate Research Centre, 60325 Frankfurt am Main, Germany; sven.winter@senckenberg.de (S.W.); barmit96@yahoo.de (B.M.)
[2] Institute for Ecology, Evolution and Diversity, Goethe University, 60439 Frankfurt am Main, Germany
[3] Giraffe Conservation Foundation, 9000 Windhoek, Namibia; julian@giraffeconservation.org (J.F.)
[4] LOEWE Centre for Translational Biodiversity Genomics, 60325 Frankfurt am Main, Germany
* Correspondence: raphael.coimbra@senckenberg.de (R.T.F.C.); axel.janke@senckenberg.de (A.J.)

Figure S1. Bayesian mitochondrial phylogeny of giraffe species and subspecies.

Figure S2. Run likelihoods per *K*.

Figure S3. Proportion of the genome associated with different HBD classes.

Table S1. Sample details for mitochondrial dataset.

Table S2. Sample details and mapping statistics for re-sequenced individuals.

References

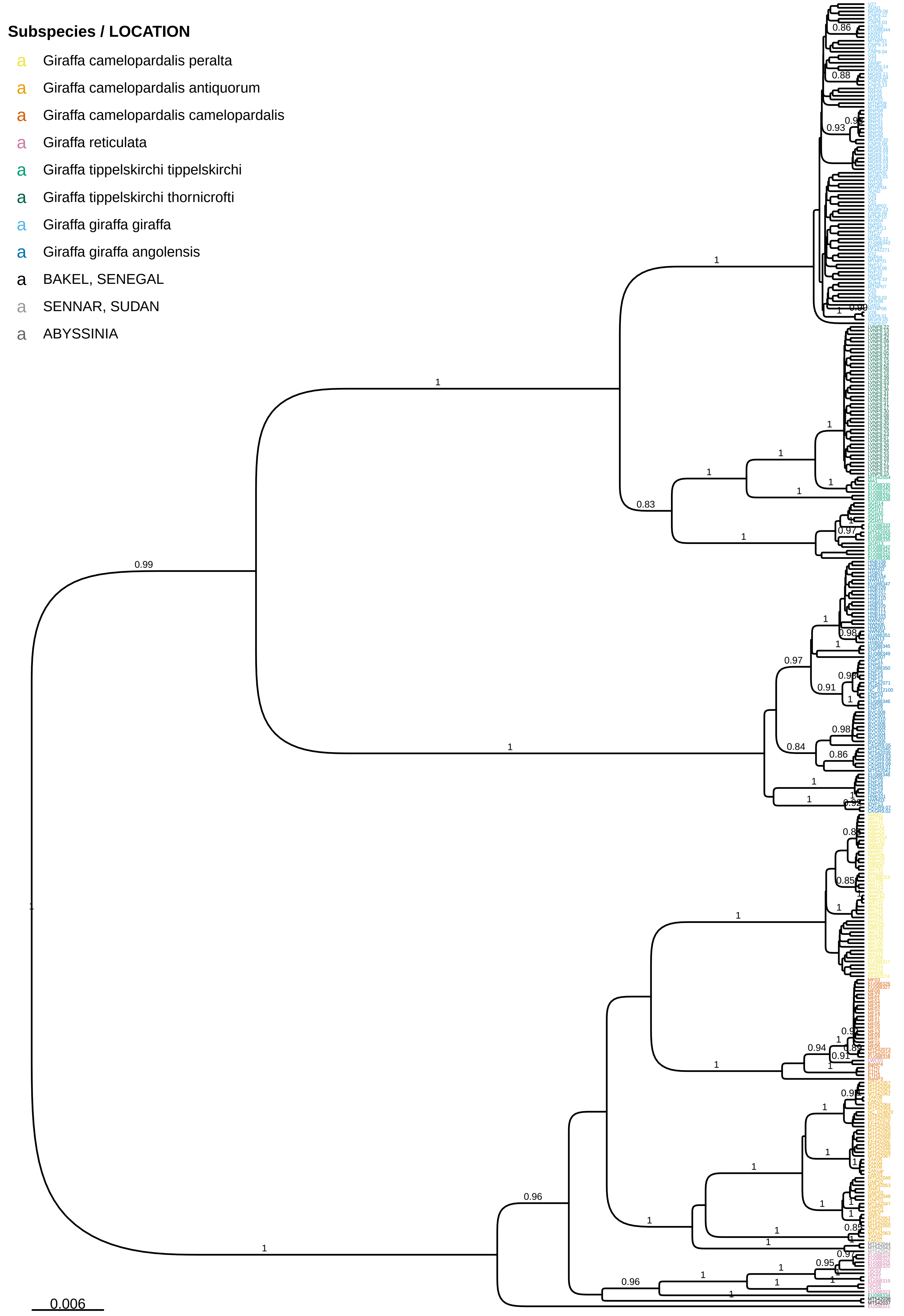
Subspecies / LOCATION
a Giraffa camelopardalis peralta
a Giraffa camelopardalis antiquorum
a Giraffa camelopardalis camelopardalis
a Giraffa reticulata
a Giraffa tippelskirchi tippelskirchi
a Giraffa tippelskirchi thornicrofti
a Giraffa giraffa giraffa
a Giraffa giraffa angolensis
a BAKEL, SENEGAL
a SENNAR, SUDAN
a ABYSSINIA
0.006

Figure S1. Bayesian mitochondrial phylogeny of giraffe species and subspecies. Mitochondrial (*Cytb* + control region) tree with 356 wild giraffe sequences representing all giraffe species and subspecies. Posterior probability (PP) support is denoted for branches with PP ≥ 0.80. The okapi was used as an outgroup (not shown).

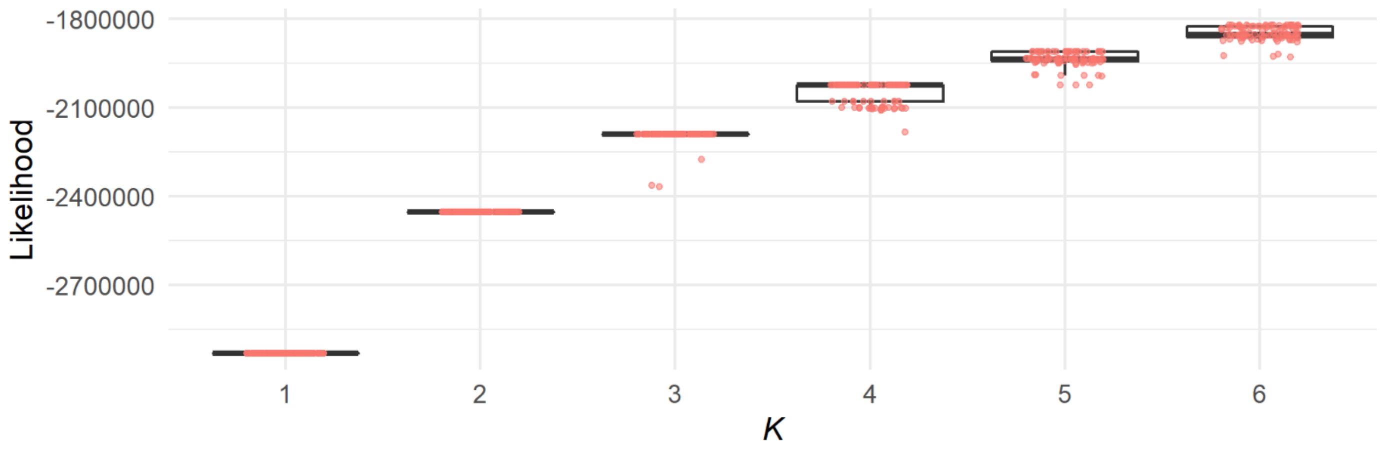

Figure S2. Run likelihoods per *K*. Median likelihood values increase with higher K values, however, at the cost of augmented statistical dispersion for $K > 3$.

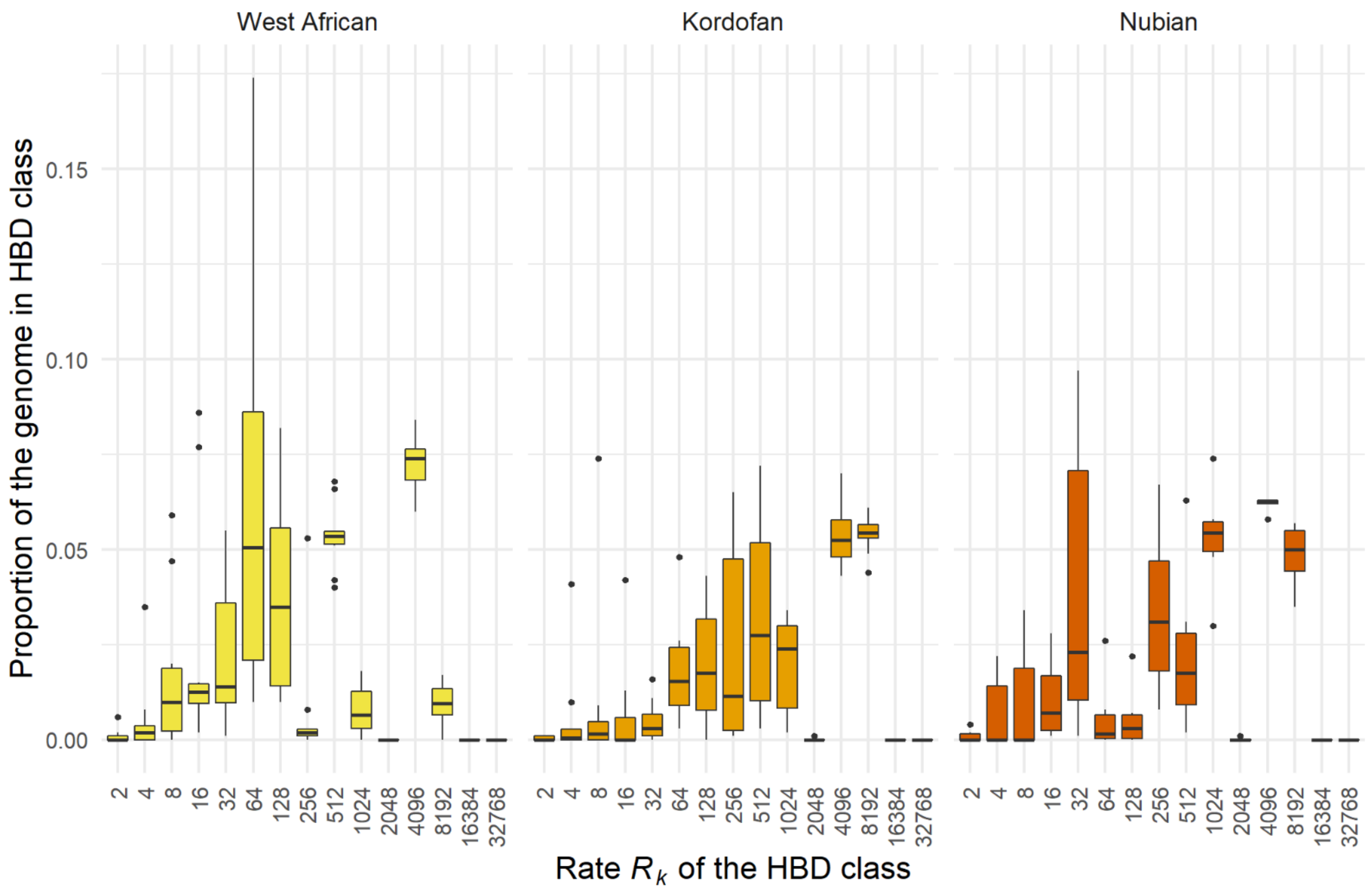

Figure S3. Proportion of the genome associated with different HBD classes. HBD classes associated with recent inbreeding ($R_k \leq 128$) were generally more abundant in West African giraffe individuals.

Table S1. Sample details for mitochondrial dataset. Sample identifier, sampling location, taxonomic classification, accession number, and original data source are shown per sequence.

Sample	Origin	Taxonomy [1]	Taxonomy [2]	Accession	Source
MNHN_A8012	Abyssinia (Ethiopia)	?	*G. camelopardalis camelopardalis*	MT542043	[2]
MHNT_OST.1996.121.2	Abyssinia (Ethiopia)	?	*G. camelopardalis camelopardalis*	MT542044	[2]
IRSNB_IG19076	Anglo-Egyptian Sudan (Sudan and South Sudan)	*G. camelopardalis antiquorum*	*G. camelopardalis antiquorum*	MT542052	[2]
CKGR9-01	Botswana: Central Kalahari Game Reserve	*G. giraffa angolensis*	*G. giraffa giraffa*	HG975196, HG975094	[3]
CKGR9-02	Botswana: Central Kalahari Game Reserve	*G. giraffa angolensis*	*G. giraffa giraffa*	HG975197, HG975095	[3]
CKGR9-03	Botswana: Central Kalahari Game Reserve	*G. giraffa angolensis*	*G. giraffa giraffa*	HG975198, HG975096	[3]
CKGR9-05	Botswana: Central Kalahari Game Reserve	*G. giraffa angolensis*	*G. giraffa giraffa*	HG975199, HG975097	[3]
CKGR9-06	Botswana: Central Kalahari Game Reserve	*G. giraffa angolensis*	*G. giraffa giraffa*	HG975200, HG975098	[3]
CKGR9-07	Botswana: Central Kalahari Game Reserve	*G. giraffa angolensis*	*G. giraffa giraffa*	HG975201, HG975099	[3]
CKGR9-08	Botswana: Central Kalahari Game Reserve	*G. giraffa angolensis*	*G. giraffa giraffa*	HG975202, HG975100	[3]
CNP9-02	Botswana: Chobe NP	*G. giraffa giraffa*	*G. giraffa wardi*	HF571215, HF571172	[4]
CNP9-03	Botswana: Chobe NP	*G. giraffa giraffa*	*G. giraffa wardi*	HF571216, HF571173	[4]
CNP9-05	Botswana: Chobe NP	*G. giraffa giraffa*	*G. giraffa wardi*	HF571217, HF571174	[4]
CNP9-04	Botswana: Chobe NP	*G. giraffa giraffa*	*G. giraffa wardi*	HG975203, HG975101	[3]
CNP9-06	Botswana: Chobe NP	*G. giraffa giraffa*	*G. giraffa wardi*	HG975204, HG975102	[3]
CNP9-07	Botswana: Chobe NP	*G. giraffa giraffa*	*G. giraffa wardi*	HG975205, HG975103	[3]
CNP9-08	Botswana: Chobe NP	*G. giraffa giraffa*	*G. giraffa wardi*	HG975206, HG975104	[3]
CNP9-09	Botswana: Chobe NP	*G. giraffa giraffa*	*G. giraffa wardi*	HG975207, HG975105	[3]
CNP9-10	Botswana: Chobe NP	*G. giraffa giraffa*	*G. giraffa wardi*	HG975208, HG975106	[3]
CNP9-12	Botswana: Chobe NP	*G. giraffa giraffa*	*G. giraffa wardi*	HG975209, HG975107	[3]
CNP9-13	Botswana: Chobe NP	*G. giraffa giraffa*	*G. giraffa wardi*	HG975210, HG975108	[3]
CNP9-14	Botswana: Chobe NP	*G. giraffa giraffa*	*G. giraffa wardi*	HG975211, HG975109	[3]
MGR9-04	Botswana: Moremi Game Reserve	*G. giraffa giraffa*	*G. giraffa wardi*	HF571210, HF571168	[4]
MGR9-11	Botswana: Moremi Game Reserve	*G. giraffa giraffa*	*G. giraffa wardi*	HF571211, HF571169	[4]
MGR9-13	Botswana: Moremi Game Reserve	*G. giraffa giraffa*	*G. giraffa wardi*	HF571212, HF571170	[4]
MGR9-01	Botswana: Moremi Game Reserve	*G. giraffa giraffa*	*G. giraffa wardi*	HG975241, HG975139	[3]
MGR9-02	Botswana: Moremi Game Reserve	*G. giraffa giraffa*	*G. giraffa wardi*	HG975242, HG975140	[3]
MGR9-03	Botswana: Moremi Game Reserve	*G. giraffa giraffa*	*G. giraffa wardi*	HG975243, HG975141	[3]
MGR9-05	Botswana: Moremi Game Reserve	*G. giraffa giraffa*	*G. giraffa wardi*	HG975244, HG975142	[3]
MGR9-06	Botswana: Moremi Game Reserve	*G. giraffa giraffa*	*G. giraffa wardi*	HG975245, HG975143	[3]
MGR9-09	Botswana: Moremi Game Reserve	*G. giraffa giraffa*	*G. giraffa wardi*	HG975246, HG975144	[3]
MGR9-12	Botswana: Moremi Game Reserve	*G. giraffa giraffa*	*G. giraffa wardi*	HG975247, HG975145	[3]
MGR9-14	Botswana: Moremi Game Reserve	*G. giraffa giraffa*	*G. giraffa wardi*	HG975248, HG975146	[3]

Sample	Origin	Taxonomy [1]	Taxonomy [2]	Accession	Source
MGR9-16	Botswana: Moremi Game Reserve	*G. giraffa giraffa*	*G. giraffa wardi*	HG975249, HG975147	[3]
MGR9-17	Botswana: Moremi Game Reserve	*G. giraffa giraffa*	*G. giraffa wardi*	HG975250, HG975148	[3]
MGR9-18	Botswana: Moremi Game Reserve	*G. giraffa giraffa*	*G. giraffa wardi*	HG975251, HG975149	[3]
MGR9-19	Botswana: Moremi Game Reserve	*G. giraffa giraffa*	*G. giraffa wardi*	HG975252, HG975150	[3]
MGR9-20	Botswana: Moremi Game Reserve	*G. giraffa giraffa*	*G. giraffa wardi*	HG975253, HG975151	[3]
NXP9-01	Botswana: Nxai Pans	*G. giraffa giraffa*	*G. giraffa wardi*	HF571213, HF571171	[4]
V22	Botswana: Vumbura Concession	*G. giraffa giraffa*	*G. giraffa wardi*	HG975266, HG975164	[3]
V23	Botswana: Vumbura Concession	*G. giraffa giraffa*	*G. giraffa wardi*	HG975267, HG975165	[3]
V24	Botswana: Vumbura Concession	*G. giraffa giraffa*	*G. giraffa wardi*	HG975268, HG975166	[3]
V25	Botswana: Vumbura Concession	*G. giraffa giraffa*	*G. giraffa wardi*	HG975269, HG975167	[3]
V26	Botswana: Vumbura Concession	*G. giraffa giraffa*	*G. giraffa wardi*	HG975270, HG975168	[3]
V27	Botswana: Vumbura Concession	*G. giraffa giraffa*	*G. giraffa wardi*	HG975271, HG975169	[3]
V28	Botswana: Vumbura Concession	*G. giraffa giraffa*	*G. giraffa wardi*	HG975272, HG975170	[3]
V30	Botswana: Vumbura Concession	*G. giraffa giraffa*	*G. giraffa wardi*	HG975273, HG975171	[3]
V31	Botswana: Vumbura Concession	*G. giraffa giraffa*	*G. giraffa wardi*	HG975274, HG975172	[3]
V32	Botswana: Vumbura Concession	*G. giraffa giraffa*	*G. giraffa wardi*	HG975275, HG975173	[3]
V33	Botswana: Vumbura Concession	*G. giraffa giraffa*	*G. giraffa wardi*	HG975276, HG975174	[3]
Waza2	Cameroon: Waza NP	*G. camelopardalis antiquorum*	*G. camelopardalis antiquorum*	MT542069	[2]
Waza3	Cameroon: Waza NP	*G. camelopardalis antiquorum*	*G. camelopardalis antiquorum*	MT542070	[2]
NC_024820	Cameroon: Waza NP	*G. camelopardalis antiquorum*	*G. camelopardalis antiquorum*	NC_024820	[5]
EF442268	Cameroon: Waza NP; Bouba Ndjida NP	*G. camelopardalis antiquorum*	*G. camelopardalis antiquorum*	EF442268	[6]
MNHN_1896-45	Cape Colony (South Africa): Orange River	*G. giraffa angolensis*	*G. giraffa giraffa*	MT542039	[2]
MNHN_A10749	Cape Colony (South Africa): Orange River	*G. giraffa angolensis*	*G. giraffa giraffa*	MT542040	[2]
MNHN_A7977	Cape Colony (South Africa): Orange River	*G. giraffa angolensis*	*G. giraffa giraffa*	MT542041	[2]
RMCA_21645M	Chad: Sarh	*G. camelopardalis antiquorum*	*G. camelopardalis antiquorum*	MT542046	[2]
EF442266	Chad: Zakouma NP	*G. camelopardalis antiquorum*	*G. camelopardalis antiquorum*	EF442266	[6]
ZNP01	Chad: Zakouma NP	*G. camelopardalis antiquorum*	*G. camelopardalis antiquorum*	HG975281, HG975179	[3]
ZED801	Chad: Zakouma NP	*G. camelopardalis antiquorum*	*G. camelopardalis antiquorum*	MT542056	[2]
ZED802	Chad: Zakouma NP	*G. camelopardalis antiquorum*	*G. camelopardalis antiquorum*	MT542057	[2]
ZED803	Chad: Zakouma NP	*G. camelopardalis antiquorum*	*G. camelopardalis antiquorum*	MT542058	[2]
ZED804	Chad: Zakouma NP	*G. camelopardalis antiquorum*	*G. camelopardalis antiquorum*	MT542059	[2]
ZED805	Chad: Zakouma NP	*G. camelopardalis antiquorum*	*G. camelopardalis antiquorum*	MT542060	[2]
ZED807	Chad: Zakouma NP	*G. camelopardalis antiquorum*	*G. camelopardalis antiquorum*	MT542061	[2]
ZED809	Chad: Zakouma NP	*G. camelopardalis antiquorum*	*G. camelopardalis antiquorum*	MT542062	[2]
ZED810	Chad: Zakouma NP	*G. camelopardalis antiquorum*	*G. camelopardalis antiquorum*	MT542063	[2]

Sample	Origin	Taxonomy [1]	Taxonomy [2]	Accession	Source
ZED812	Chad: Zakouma NP	*G. camelopardalis antiquorum*	*G. camelopardalis antiquorum*	MT542064	[2]
ZED813	Chad: Zakouma NP	*G. camelopardalis antiquorum*	*G. camelopardalis antiquorum*	MT542065	[2]
ZED817	Chad: Zakouma NP	*G. camelopardalis antiquorum*	*G. camelopardalis antiquorum*	MT542066	[2]
ZED818	Chad: Zakouma NP	*G. camelopardalis antiquorum*	*G. camelopardalis antiquorum*	MT542067	[2]
ZED819	Chad: Zakouma NP	*G. camelopardalis antiquorum*	*G. camelopardalis antiquorum*	MT542068	[2]
ZAK01	Chad: Zakouma NP	*G. camelopardalis antiquorum*	*G. camelopardalis antiquorum*	OK558563, OK558591	This study
ZAK02	Chad: Zakouma NP	*G. camelopardalis antiquorum*	*G. camelopardalis antiquorum*	OK558564	This study
ZAK03	Chad: Zakouma NP	*G. camelopardalis antiquorum*	*G. camelopardalis antiquorum*	OK558565, OK558592	This study
ZAK04	Chad: Zakouma NP	*G. camelopardalis antiquorum*	*G. camelopardalis antiquorum*	OK558566, OK558593	This study
ZAK05	Chad: Zakouma NP	*G. camelopardalis antiquorum*	*G. camelopardalis antiquorum*	OK558567, OK558594	This study
ZAK06	Chad: Zakouma NP	*G. camelopardalis antiquorum*	*G. camelopardalis antiquorum*	OK558568, OK558595	This study
ZAK07	Chad: Zakouma NP	*G. camelopardalis antiquorum*	*G. camelopardalis antiquorum*	OK558569, OK558596	This study
ZAK08	Chad: Zakouma NP	*G. camelopardalis antiquorum*	*G. camelopardalis antiquorum*	OK558570, OK558597	This study
ZAKcar	Chad: Zakouma NP	*G. camelopardalis antiquorum*	*G. camelopardalis antiquorum*	OK558571, OK558598	This study
RMCA_25672M	DR Congo: Gangala	*G. camelopardalis antiquorum*	*G. camelopardalis antiquorum*	MT542047	[2]
RMCA_25673M	DR Congo: Gangala	*G. camelopardalis antiquorum*	*G. camelopardalis antiquorum*	MT542048	[2]
GNP01	DR Congo: Garamba NP	*G. camelopardalis antiquorum*	*G. camelopardalis antiquorum*	HG975229, HG975127	[3]
GNP02	DR Congo: Garamba NP	*G. camelopardalis antiquorum*	*G. camelopardalis antiquorum*	HG975230, HG975128	[3]
GNP04	DR Congo: Garamba NP	*G. camelopardalis antiquorum*	*G. camelopardalis antiquorum*	HG975231, HG975129	[3]
GNP03	DR Congo: Garamba NP	*G. camelopardalis antiquorum*	*G. camelopardalis antiquorum*	MG257950, MG262283	[7]
GNP05	DR Congo: Garamba NP	*G. camelopardalis antiquorum*	*G. camelopardalis antiquorum*	MG257951, MG262284	[7]
RMCA_83.006M0553	DR Congo: Garamba NP	*G. camelopardalis antiquorum*	*G. camelopardalis antiquorum*	MT542049	[2]
RMCA_3748M	DR Congo: Kapili	*G. camelopardalis antiquorum*	*G. camelopardalis antiquorum*	MT542050	[2]
RMCA_5956M	DR Congo: northeast Uélé	*G. camelopardalis antiquorum*	*G. camelopardalis antiquorum*	MT542053	[2]
ETH1	Ethiopia: Gambella NP	*G. camelopardalis camelopardalis*	*G. camelopardalis rothschildi*	LT628370, LT628397	[1]
ETH2	Ethiopia: Gambella NP	*G. camelopardalis camelopardalis*	*G. camelopardalis rothschildi*	MG257948, MG262281	[7]
ETH3	Ethiopia: Gambella NP	*G. camelopardalis camelopardalis*	*G. camelopardalis rothschildi*	MG257949, MG262282	[7]
EU088321	Kenya	*G. reticulata*	*G. camelopardalis reticulata*	EU088321	[8]
MNHN_1913-523	Kenya	*G. tippelskirchi tippelskirchi*	*G. tippelskirchi tippelskirchi*	MT542054	[2]
EU088333	Kenya: Athi River Ranch	*G. tippelskirchi tippelskirchi*	*G. tippelskirchi tippelskirchi*	EU088333	[8]
EU088334	Kenya: Athi River Ranch	*G. tippelskirchi tippelskirchi*	*G. tippelskirchi tippelskirchi*	EU088334	[8]
EU088329	Kenya: Athi River Ranch; Chyulu Hills<br>Tanzania: Lake Naivasha; Manyara NP; Tarangire NP	*G. tippelskirchi tippelskirchi*	*G. tippelskirchi tippelskirchi*	EU088329	[8]
ROTH_B	Kenya: Baringo	*G. camelopardalis camelopardalis*	*G. camelopardalis rothschildi*	MT542072	[2]
EU088336	Kenya: Chyulu Hills	*G. tippelskirchi tippelskirchi*	*G. tippelskirchi tippelskirchi*	EU088336	[8]

Sample	Origin	Taxonomy [1]	Taxonomy [2]	Accession	Source
EU088331	Kenya: Chyulu Hills<br>Tanzania: Lake Naivasha; Manyara NP; Tarangire NP	*G. tippelskirchi tippelskirchi*	*G. tippelskirchi tippelskirchi*	EU088331	[8]
ROTH_D	Kenya: Elmenteita	*G. camelopardalis camelopardalis*	*G. camelopardalis rothschildi*	MT542073	[2]
ISC03	Kenya: Ishqbini Conservancy	*G. reticulata*	*G. camelopardalis reticulata*	KY865127, KY865164	[9]
ISC04	Kenya: Ishqbini Conservancy	*G. reticulata*	*G. camelopardalis reticulata*	KY865128, KY865165	[9]
ISC05	Kenya: Ishqbini Conservancy	*G. reticulata*	*G. camelopardalis reticulata*	KY865129, KY865166	[9]
ISC08	Kenya: Ishqbini Conservancy	*G. reticulata*	*G. camelopardalis reticulata*	KY865130, KY865167	[9]
ISC01	Kenya: Ishqbini Conservancy	*G. reticulata*	*G. camelopardalis reticulata*	MT605028	[10]
LWC01	Kenya: Loisaba Wildlife Conservancy	*G. reticulata*	*G. camelopardalis reticulata*	MG257952, MG262285	[7]
MA1	Kenya: Masai Mara National Reserve	*G. tippelskirchi tippelskirchi*	*G. tippelskirchi tippelskirchi*	MT605044	[10]
EU088319	Kenya: Meru NP; Ol Jogi, Pyramid, Sweetwaters - Laikipia; Samburu NR	*G. reticulata*	*G. camelopardalis reticulata*	EU088319	[8]
EU088322	Kenya: Meru NP; Ol Jogi, Pyramid, Sweetwaters - Laikipia; Samburu NR	*G. reticulata*	*G. camelopardalis reticulata*	EU088322	[8]
EU088327	Kenya: Nakuru NP; Ruma NP	*G. camelopardalis camelopardalis*	*G. camelopardalis rothschildi*	EU088327	[8]
EU088328	Kenya: Nakuru NP; Ruma NP	*G. camelopardalis camelopardalis*	*G. camelopardalis rothschildi*	EU088328	[8]
EU088326	Kenya: Nakuru NP; Ruma NP<br>Uganda: Murchison Falls NP	*G. camelopardalis camelopardalis*	*G. camelopardalis rothschildi*	EU088326	[8]
EU088320	Kenya: Ol Jogi, Pyramid, Sweetwaters - Laikipia; Samburu NR	*G. reticulata*	*G. camelopardalis reticulata*	EU088320	[8]
EU088324	Kenya: Pyramid, Laikipia	*G. reticulata*	*G. camelopardalis reticulata*	EU088324	[8]
EU088325	Kenya: Pyramid, Laikipia	*G. reticulata*	*G. camelopardalis reticulata*	EU088325	[8]
RMCA_2128M	Kenya: Serengeti-Mara	*G. tippelskirchi tippelskirchi*	*G. tippelskirchi tippelskirchi*	MT542055	[2]
EU088323	Kenya: Sweetwaters, Laikipia	*G. reticulata*	*G. camelopardalis reticulata*	EU088323	[8]
RMCA_767M	Lado Enclave (Northwest Uganda and South Sudan)	*G. camelopardalis antiquorum*	*G. camelopardalis antiquorum*	MT542051	[2]
GH01	Malawi: Game Haven Lodge	*G. giraffa giraffa*	*G. giraffa wardi*	MH782194, MH782208	[11]
GH02	Malawi: Game Haven Lodge	*G. giraffa giraffa*	*G. giraffa wardi*	MH782195, MH782209	[11]
NyP01	Malawi: Nyala Game Park	*G. giraffa giraffa*	*G. giraffa wardi*	MH782196, MH782210	[11]
NyP02	Malawi: Nyala Game Park	*G. giraffa giraffa*	*G. giraffa wardi*	MH782197, MH782211	[11]
NyP03	Malawi: Nyala Game Park	*G. giraffa giraffa*	*G. giraffa wardi*	MH782198, MH782212	[11]
NyP04	Malawi: Nyala Game Park	*G. giraffa giraffa*	*G. giraffa wardi*	MH782199, MH782213	[11]
NyP05	Malawi: Nyala Game Park	*G. giraffa giraffa*	*G. giraffa wardi*	MH782200, MH782214	[11]
NyP06	Malawi: Nyala Game Park	*G. giraffa giraffa*	*G. giraffa wardi*	MH782201, MH782215	[11]
NyP07	Malawi: Nyala Game Park	*G. giraffa giraffa*	*G. giraffa wardi*	MH782202, MH782216	[11]
NyP08	Malawi: Nyala Game Park	*G. giraffa giraffa*	*G. giraffa wardi*	MH782203, MH782217	[11]
NyP09	Malawi: Nyala Game Park	*G. giraffa giraffa*	*G. giraffa wardi*	MH782204, MH782218	[11]
NyP10	Malawi: Nyala Game Park	*G. giraffa giraffa*	*G. giraffa wardi*	MH782205, MH782219	[11]
NyP11	Malawi: Nyala Game Park	*G. giraffa giraffa*	*G. giraffa wardi*	MH782206, MH782220	[11]
NyP12	Malawi: Nyala Game Park	*G. giraffa giraffa*	*G. giraffa wardi*	MH782207, MH782221	[11]

Sample	Origin	Taxonomy [1]	Taxonomy [2]	Accession	Source
NC_012100	?	*G. giraffa angolensis*	*G. giraffa giraffa*	NC_012100	Unpublished
BNP01	Namibia: Bwabwata NP	*G. giraffa giraffa*	*G. giraffa wardi*	HG975189, HG975087	[3]
BNP04	Namibia: Bwabwata NP	*G. giraffa giraffa*	*G. giraffa wardi*	HG975190, HG975088	[3]
BNP05	Namibia: Bwabwata NP	*G. giraffa giraffa*	*G. giraffa wardi*	HG975191, HG975089	[3]
BNP06	Namibia: Bwabwata NP	*G. giraffa giraffa*	*G. giraffa wardi*	HG975192, HG975090	[3]
BNP07	Namibia: Bwabwata NP	*G. giraffa giraffa*	*G. giraffa wardi*	HG975193, HG975091	[3]
BNP08	Namibia: Bwabwata NP	*G. giraffa giraffa*	*G. giraffa wardi*	HG975194, HG975092	[3]
BNP09	Namibia: Bwabwata NP	*G. giraffa giraffa*	*G. giraffa wardi*	HG975195, HG975093	[3]
BNP02	Namibia: Bwabwata NP	*G. giraffa giraffa*	*G. giraffa wardi*	MT605050	[10]
EU088345	Namibia: Etosha NP	*G. giraffa angolensis*	*G. giraffa giraffa*	EU088345	[8]
EU088348	Namibia: Etosha NP	*G. giraffa angolensis*	*G. giraffa giraffa*	EU088348	[8]
EU088349	Namibia: Etosha NP	*G. giraffa angolensis*	*G. giraffa giraffa*	EU088349	[8]
ENP01	Namibia: Etosha NP	*G. giraffa angolensis*	*G. giraffa giraffa*	HG975212, HG975110	[3]
ENP04	Namibia: Etosha NP	*G. giraffa angolensis*	*G. giraffa giraffa*	HG975213, HG975111	[3]
ENP05	Namibia: Etosha NP	*G. giraffa angolensis*	*G. giraffa giraffa*	HG975214, HG975112	[3]
ENP06	Namibia: Etosha NP	*G. giraffa angolensis*	*G. giraffa giraffa*	HG975215, HG975113	[3]
ENP07	Namibia: Etosha NP	*G. giraffa angolensis*	*G. giraffa giraffa*	HG975216, HG975114	[3]
ENP08	Namibia: Etosha NP	*G. giraffa angolensis*	*G. giraffa giraffa*	HG975217, HG975115	[3]
ENP11	Namibia: Etosha NP	*G. giraffa angolensis*	*G. giraffa giraffa*	HG975218, HG975116	[3]
ENP12	Namibia: Etosha NP	*G. giraffa angolensis*	*G. giraffa giraffa*	HG975219, HG975117	[3]
ENP13	Namibia: Etosha NP	*G. giraffa angolensis*	*G. giraffa giraffa*	HG975220, HG975118	[3]
ENP14	Namibia: Etosha NP	*G. giraffa angolensis*	*G. giraffa giraffa*	HG975221, HG975119	[3]
ENP15	Namibia: Etosha NP	*G. giraffa angolensis*	*G. giraffa giraffa*	HG975222, HG975120	[3]
ENP16	Namibia: Etosha NP	*G. giraffa angolensis*	*G. giraffa giraffa*	HG975223, HG975121	[3]
ENP17	Namibia: Etosha NP	*G. giraffa angolensis*	*G. giraffa giraffa*	HG975224, HG975122	[3]
ENP18	Namibia: Etosha NP	*G. giraffa angolensis*	*G. giraffa giraffa*	HG975225, HG975123	[3]
ENP19	Namibia: Etosha NP	*G. giraffa angolensis*	*G. giraffa giraffa*	HG975226, HG975124	[3]
ENP20	Namibia: Etosha NP	*G. giraffa angolensis*	*G. giraffa giraffa*	HG975227, HG975125	[3]
ENP21	Namibia: Etosha NP	*G. giraffa angolensis*	*G. giraffa giraffa*	HG975228, HG975126	[3]
EU088346	Namibia: Etosha NP; Kamanjab	*G. giraffa angolensis*	*G. giraffa giraffa*	EU088346	[8]
HNB001	Namibia: Hoanib River catchment	*G. giraffa angolensis*	*G. giraffa giraffa*	KY865111, KY865148	[9]
HNB101	Namibia: Hoanib River catchment	*G. giraffa angolensis*	*G. giraffa giraffa*	KY865112, KY865149	[9]
HNB102	Namibia: Hoanib River catchment	*G. giraffa angolensis*	*G. giraffa giraffa*	KY865113, KY865150	[9]
HNB103	Namibia: Hoanib River catchment	*G. giraffa angolensis*	*G. giraffa giraffa*	KY865114, KY865151	[9]
HNB104	Namibia: Hoanib River catchment	*G. giraffa angolensis*	*G. giraffa giraffa*	KY865115, KY865152	[9]

Sample	Origin	Taxonomy [1]	Taxonomy [2]	Accession	Source
HNB105	Namibia: Hoanib River catchment	*G. giraffa angolensis*	*G. giraffa giraffa*	KY865116, KY865153	[9]
HNB106	Namibia: Hoanib River catchment	*G. giraffa angolensis*	*G. giraffa giraffa*	KY865117, KY865154	[9]
HNB107	Namibia: Hoanib River catchment	*G. giraffa angolensis*	*G. giraffa giraffa*	KY865118, KY865155	[9]
HNB108	Namibia: Hoanib River catchment	*G. giraffa angolensis*	*G. giraffa giraffa*	KY865119, KY865156	[9]
HNB109	Namibia: Hoanib River catchment	*G. giraffa angolensis*	*G. giraffa giraffa*	KY865120, KY865157	[9]
HNB110	Namibia: Hoanib River catchment	*G. giraffa angolensis*	*G. giraffa giraffa*	KY865121, KY865158	[9]
HNB111	Namibia: Hoanib River catchment	*G. giraffa angolensis*	*G. giraffa giraffa*	KY865122, KY865159	[9]
HNB112	Namibia: Hoanib River catchment	*G. giraffa angolensis*	*G. giraffa giraffa*	KY865123, KY865160	[9]
EU088347	Namibia: Hoanib River catchment; Khumib River catchment	*G. giraffa angolensis*	*G. giraffa giraffa*	EU088347	[8]
EU088350	Namibia: Hoarusib River catchment	*G. giraffa angolensis*	*G. giraffa giraffa*	EU088350	[8]
EU088351	Namibia: Hoarusib River catchment	*G. giraffa angolensis*	*G. giraffa giraffa*	EU088351	[8]
HSB01	Namibia: Hoarusib River catchment	*G. giraffa angolensis*	*G. giraffa giraffa*	KY865124, KY865161	[9]
HSB03	Namibia: Hoarusib River catchment	*G. giraffa angolensis*	*G. giraffa giraffa*	KY865125, KY865162	[9]
HSB04	Namibia: Hoarusib River catchment	*G. giraffa angolensis*	*G. giraffa giraffa*	KY865126, KY865163	[9]
EU088317	Niger: Harikanassou region	*G. camelopardalis peralta*	*G. camelopardalis peralta*	EU088317	[8]
EU088318	Niger: Harikanassou region	*G. camelopardalis peralta*	*G. camelopardalis peralta*	EU088318	[8]
WA117	Niger: Kouré plateau	*G. camelopardalis peralta*	*G. camelopardalis peralta*	HG975277, HG975175	[3]
WA026	Niger: Kouré plateau	*G. camelopardalis peralta*	*G. camelopardalis peralta*	HG975278, HG975176	[3]
WA036	Niger: Kouré plateau	*G. camelopardalis peralta*	*G. camelopardalis peralta*	HG975279, HG975177	[3]
WA606	Niger: Kouré plateau	*G. camelopardalis peralta*	*G. camelopardalis peralta*	HG975280, HG975178	[3]
WA609	Niger: Kouré plateau	*G. camelopardalis peralta*	*G. camelopardalis peralta*	HG975282, HG975180	[3]
WA612	Niger: Kouré plateau	*G. camelopardalis peralta*	*G. camelopardalis peralta*	HG975283, HG975181	[3]
WA614	Niger: Kouré plateau	*G. camelopardalis peralta*	*G. camelopardalis peralta*	HG975284, HG975182	[3]
WA619	Niger: Kouré plateau	*G. camelopardalis peralta*	*G. camelopardalis peralta*	HG975285, HG975183	[3]
WA621	Niger: Kouré plateau	*G. camelopardalis peralta*	*G. camelopardalis peralta*	HG975286, HG975184	[3]
WA622	Niger: Kouré plateau	*G. camelopardalis peralta*	*G. camelopardalis peralta*	HG975287, HG975185	[3]
WA623	Niger: Kouré plateau	*G. camelopardalis peralta*	*G. camelopardalis peralta*	HG975288, HG975186	[3]
WA628	Niger: Kouré plateau	*G. camelopardalis peralta*	*G. camelopardalis peralta*	HG975289, HG975187	[3]
WA631	Niger: Kouré plateau	*G. camelopardalis peralta*	*G. camelopardalis peralta*	HG975290, HG975188	[3]
WA700	Niger: Kouré plateau	*G. camelopardalis peralta*	*G. camelopardalis peralta*	MG257964, MG262297	[7]
WA705	Niger: Kouré plateau	*G. camelopardalis peralta*	*G. camelopardalis peralta*	MG257965, MG262298	[7]
WA707	Niger: Kouré plateau	*G. camelopardalis peralta*	*G. camelopardalis peralta*	MG257966, MG262299	[7]
WA708	Niger: Kouré plateau	*G. camelopardalis peralta*	*G. camelopardalis peralta*	MG257967, MG262300	[7]
WA720	Niger: Kouré plateau	*G. camelopardalis peralta*	*G. camelopardalis peralta*	MG257968, MG262301	[7]
WA733	Niger: Kouré plateau	*G. camelopardalis peralta*	*G. camelopardalis peralta*	MT605013	[10]

Sample	Origin	Taxonomy [1]	Taxonomy [2]	Accession	Source
WA746	Niger: Kouré plateau	*G. camelopardalis peralta*	*G. camelopardalis peralta*	MT605014	[10]
WA806	Niger: Kouré plateau	*G. camelopardalis peralta*	*G. camelopardalis peralta*	MT605015	[10]
WA808	Niger: Kouré plateau	*G. camelopardalis peralta*	*G. camelopardalis peralta*	MT605016	[10]
Niger01a	Niger: Kouré plateau	*G. camelopardalis peralta*	*G. camelopardalis peralta*	OK558543, OK558572	This study
Niger02	Niger: Kouré plateau	*G. camelopardalis peralta*	*G. camelopardalis peralta*	OK558544	This study
Niger03	Niger: Kouré plateau	*G. camelopardalis peralta*	*G. camelopardalis peralta*	OK558545, OK558573	This study
Niger04	Niger: Kouré plateau	*G. camelopardalis peralta*	*G. camelopardalis peralta*	OK558546, OK558574	This study
Niger05	Niger: Kouré plateau	*G. camelopardalis peralta*	*G. camelopardalis peralta*	OK558547, OK558575	This study
Niger06	Niger: Kouré plateau	*G. camelopardalis peralta*	*G. camelopardalis peralta*	OK558548, OK558576	This study
Niger07	Niger: Kouré plateau	*G. camelopardalis peralta*	*G. camelopardalis peralta*	OK558549, OK558577	This study
Niger08	Niger: Kouré plateau	*G. camelopardalis peralta*	*G. camelopardalis peralta*	OK558550, OK558578	This study
Niger09	Niger: Kouré plateau	*G. camelopardalis peralta*	*G. camelopardalis peralta*	OK558551, OK558579	This study
Niger10	Niger: Kouré plateau	*G. camelopardalis peralta*	*G. camelopardalis peralta*	OK558552, OK558580	This study
Niger11	Niger: Kouré plateau	*G. camelopardalis peralta*	*G. camelopardalis peralta*	OK558553, OK558581	This study
Niger12	Niger: Kouré plateau	*G. camelopardalis peralta*	*G. camelopardalis peralta*	OK558554, OK558582	This study
WA732	Niger: Kouré plateau	*G. camelopardalis peralta*	*G. camelopardalis peralta*	OK558555, OK558583	This study
WA736	Niger: Kouré plateau	*G. camelopardalis peralta*	*G. camelopardalis peralta*	OK558556, OK558584	This study
WA741	Niger: Kouré plateau	*G. camelopardalis peralta*	*G. camelopardalis peralta*	OK558557, OK558585	This study
WA801	Niger: Kouré plateau	*G. camelopardalis peralta*	*G. camelopardalis peralta*	OK558558, OK558586	This study
WA804	Niger: Kouré plateau	*G. camelopardalis peralta*	*G. camelopardalis peralta*	OK558559, OK558587	This study
WA805	Niger: Kouré plateau	*G. camelopardalis peralta*	*G. camelopardalis peralta*	OK558560, OK558588	This study
WA807	Niger: Kouré plateau	*G. camelopardalis peralta*	*G. camelopardalis peralta*	OK558561, OK558589	This study
WA811	Niger: Kouré plateau	*G. camelopardalis peralta*	*G. camelopardalis peralta*	OK558562, OK558590	This study
EF442274	Niger: Kouré plateau; Niamey-Dosso Road	*G. camelopardalis peralta*	*G. camelopardalis peralta*	EF442274	[6]
ZMB_48222	Nigeria: Dikoa	*G. camelopardalis antiquorum*	*G. camelopardalis antiquorum*	MT542045	[2]
NWN02	Northwestern Namibia	*G. giraffa angolensis*	*G. giraffa giraffa*	KY865131, KY865168	[9]
NWN03	Northwestern Namibia	*G. giraffa angolensis*	*G. giraffa giraffa*	KY865132, KY865169	[9]
NWN04	Northwestern Namibia	*G. giraffa angolensis*	*G. giraffa giraffa*	KY865133, KY865170	[9]
NWN06	Northwestern Namibia	*G. giraffa angolensis*	*G. giraffa giraffa*	KY865134, KY865171	[9]
NWN07	Northwestern Namibia	*G. giraffa angolensis*	*G. giraffa giraffa*	KY865135, KY865172	[9]
NWN12	Northwestern Namibia	*G. giraffa angolensis*	*G. giraffa giraffa*	KY865136, KY865173	[9]
NWN13	Northwestern Namibia	*G. giraffa angolensis*	*G. giraffa giraffa*	KY865137, KY865174	[9]
MNHN_A10753	Senegal: Bakel	?	*G. camelopardalis senegalensis*	MT542037	[2]
MNHN_A10617	Senegal: Bakel	?	*G. camelopardalis senegalensis*	MT542038	[2]
SAF6	South Africa: Augrabies Falls	*G. giraffa angolensis*	*G. giraffa giraffa*	MT542071	[2]

**Sample**	**Origin**	**Taxonomy** [1]	**Taxonomy** [2]	**Accession**	**Source**
KKR01	South Africa: Khamab Kalahari Reserve	*G. giraffa giraffa*	*G. giraffa wardi*	HG975254, HG975152	[3]
KKR02	South Africa: Khamab Kalahari Reserve	*G. giraffa giraffa*	*G. giraffa wardi*	HG975255, HG975153	[3]
KKR03	South Africa: Khamab Kalahari Reserve	*G. giraffa giraffa*	*G. giraffa wardi*	HG975256, HG975154	[3]
KKR04	South Africa: Khamab Kalahari Reserve	*G. giraffa giraffa*	*G. giraffa wardi*	HG975257, HG975155	[3]
KKR06	South Africa: Khamab Kalahari Reserve	*G. giraffa giraffa*	*G. giraffa wardi*	HG975258, HG975156	[3]
KKR07	South Africa: Khamab Kalahari Reserve	*G. giraffa giraffa*	*G. giraffa wardi*	HG975259, HG975157	[3]
KKR08	South Africa: Khamab Kalahari Reserve	*G. giraffa giraffa*	*G. giraffa wardi*	MT605052	[10]
EU088344	South Africa: Kruger NP	*G. giraffa giraffa*	*G. giraffa wardi*	EU088344	[8]
EU088343	South Africa: Kruger NP<br>Zimbabwe: Serondella	*G. giraffa giraffa*	*G. giraffa wardi*	EU088343	[8]
EF442271	South Africa: Phalaborwa, Limpopo	*G. giraffa giraffa*	*G. giraffa wardi*	EF442271	[6]
BaNP3	South Sudan: Badingilo NP	*G. camelopardalis camelopardalis*	*G. camelopardalis rothschildi*	LT628368, LT628395	[1]
BaNP4	South Sudan: Badingilo NP	*G. camelopardalis camelopardalis*	*G. camelopardalis rothschildi*	LT628369, LT628396	[1]
SNR1	South Sudan: Shambe NP	*G. camelopardalis antiquorum*	*G. camelopardalis antiquorum*	LT628389, LT628416	[1]
SNR2	South Sudan: Shambe NP	*G. camelopardalis antiquorum*	*G. camelopardalis antiquorum*	LT628390, LT628417	[1]
MNHN_1845-211_Zarafa	Sudan: Sennar	?	*G. camelopardalis camelopardalis*	MT542042	[2]
SGR01	Tanzania: Selous Game Reserve	*G. tippelskirchi tippelskirchi*	*G. tippelskirchi tippelskirchi*	HG975260, HG975158	[3]
SGR05	Tanzania: Selous Game Reserve	*G. tippelskirchi tippelskirchi*	*G. tippelskirchi tippelskirchi*	HG975261, HG975159	[3]
SGR11	Tanzania: Selous Game Reserve	*G. tippelskirchi tippelskirchi*	*G. tippelskirchi tippelskirchi*	HG975262, HG975160	[3]
SGR12	Tanzania: Selous Game Reserve	*G. tippelskirchi tippelskirchi*	*G. tippelskirchi tippelskirchi*	HG975263, HG975161	[3]
SGR13	Tanzania: Selous Game Reserve	*G. tippelskirchi tippelskirchi*	*G. tippelskirchi tippelskirchi*	HG975264, HG975162	[3]
SGR14	Tanzania: Selous Game Reserve	*G. tippelskirchi tippelskirchi*	*G. tippelskirchi tippelskirchi*	HG975265, HG975163	[3]
SGR07	Tanzania: Selous Game Reserve	*G. tippelskirchi tippelskirchi*	*G. tippelskirchi tippelskirchi*	MT605047	[10]
EU088340	Tanzania: Serengeti NP (Lobo)	*G. tippelskirchi tippelskirchi*	*G. tippelskirchi tippelskirchi*	EU088340	[8]
EU088330	Tanzania: Serengeti NP (Lobo, Ndutu, Seronera); Manyara NP	*G. tippelskirchi tippelskirchi*	*G. tippelskirchi tippelskirchi*	EU088330	[8]
EU088338	Tanzania: Serengeti NP (Ndutu, Ngorongoro)	*G. tippelskirchi tippelskirchi*	*G. tippelskirchi tippelskirchi*	EU088338	[8]
EU088337	Tanzania: Serengeti NP (Ndutu, Seronera)	*G. tippelskirchi tippelskirchi*	*G. tippelskirchi tippelskirchi*	EU088337	[8]
EU088339	Tanzania: Serengeti NP (Varicho)	*G. tippelskirchi tippelskirchi*	*G. tippelskirchi tippelskirchi*	EU088339	[8]
EU088332	Tanzania: Tarangire NP	*G. tippelskirchi tippelskirchi*	*G. tippelskirchi tippelskirchi*	EU088332	[8]
EU088341	Tanzania: Tarangire NP	*G. tippelskirchi tippelskirchi*	*G. tippelskirchi tippelskirchi*	EU088341	[8]
EU088342	Tanzania: Tarangire NP	*G. tippelskirchi tippelskirchi*	*G. tippelskirchi tippelskirchi*	EU088342	[8]
MF01	Uganda: Murchison Falls NP	*G. camelopardalis camelopardalis*	*G. camelopardalis rothschildi*	HG975232, HG975130	[3]
MF02	Uganda: Murchison Falls NP	*G. camelopardalis camelopardalis*	*G. camelopardalis rothschildi*	HG975233, HG975131	[3]
MF03	Uganda: Murchison Falls NP	*G. camelopardalis camelopardalis*	*G. camelopardalis rothschildi*	HG975234, HG975132	[3]
MF04	Uganda: Murchison Falls NP	*G. camelopardalis camelopardalis*	*G. camelopardalis rothschildi*	HG975235, HG975133	[3]

Sample	Origin	Taxonomy [1]	Taxonomy [2]	Accession	Source
MF05	Uganda: Murchison Falls NP	*G. camelopardalis camelopardalis*	*G. camelopardalis rothschildi*	HG975236, HG975134	[3]
MF06	Uganda: Murchison Falls NP	*G. camelopardalis camelopardalis*	*G. camelopardalis rothschildi*	HG975237, HG975135	[3]
MF07	Uganda: Murchison Falls NP	*G. camelopardalis camelopardalis*	*G. camelopardalis rothschildi*	HG975238, HG975136	[3]
MF08	Uganda: Murchison Falls NP	*G. camelopardalis camelopardalis*	*G. camelopardalis rothschildi*	HG975239, HG975137	[3]
MF09	Uganda: Murchison Falls NP	*G. camelopardalis camelopardalis*	*G. camelopardalis rothschildi*	HG975240, HG975138	[3]
MF11	Uganda: Murchison Falls NP	*G. camelopardalis camelopardalis*	*G. camelopardalis rothschildi*	MG257953, MG262286	[7]
MF13	Uganda: Murchison Falls NP	*G. camelopardalis camelopardalis*	*G. camelopardalis rothschildi*	MG257954, MG262287	[7]
MF14	Uganda: Murchison Falls NP	*G. camelopardalis camelopardalis*	*G. camelopardalis rothschildi*	MG257955, MG262288	[7]
MF15	Uganda: Murchison Falls NP	*G. camelopardalis camelopardalis*	*G. camelopardalis rothschildi*	MG257956, MG262289	[7]
MF16	Uganda: Murchison Falls NP	*G. camelopardalis camelopardalis*	*G. camelopardalis rothschildi*	MG257957, MG262290	[7]
MF17	Uganda: Murchison Falls NP	*G. camelopardalis camelopardalis*	*G. camelopardalis rothschildi*	MG257958, MG262291	[7]
MF24	Uganda: Murchison Falls NP	*G. camelopardalis camelopardalis*	*G. camelopardalis rothschildi*	MG257959, MG262292	[7]
MF22	Uganda: Murchison Falls NP	*G. camelopardalis camelopardalis*	*G. camelopardalis rothschildi*	MT605026	[10]
MTNP01	Zambia: Mosi-oa-Tunya NP	*G. giraffa giraffa*	*G. giraffa wardi*	LT628371, LT628398	[1]
MTNP02	Zambia: Mosi-oa-Tunya NP	*G. giraffa giraffa*	*G. giraffa wardi*	LT628372, LT628399	[1]
MTNP03	Zambia: Mosi-oa-Tunya NP	*G. giraffa giraffa*	*G. giraffa wardi*	LT628373, LT628400	[1]
MTNP04	Zambia: Mosi-oa-Tunya NP	*G. giraffa giraffa*	*G. giraffa wardi*	LT628374, LT628401	[1]
MTNP05	Zambia: Mosi-oa-Tunya NP	*G. giraffa giraffa*	*G. giraffa wardi*	LT628375, LT628402	[1]
MTNP06	Zambia: Mosi-oa-Tunya NP	*G. giraffa giraffa*	*G. giraffa wardi*	LT628376, LT628403	[1]
MTNP07	Zambia: Mosi-oa-Tunya NP	*G. giraffa giraffa*	*G. giraffa wardi*	LT628377, LT628404	[1]
MTNP08	Zambia: Mosi-oa-Tunya NP	*G. giraffa giraffa*	*G. giraffa wardi*	LT628378, LT628405	[1]
MTNP09	Zambia: Mosi-oa-Tunya NP	*G. giraffa giraffa*	*G. giraffa wardi*	LT628379, LT628406	[1]
MTNP10	Zambia: Mosi-oa-Tunya NP	*G. giraffa giraffa*	*G. giraffa wardi*	LT628380, LT628407	[1]
MTNP11	Zambia: Mosi-oa-Tunya NP	*G. giraffa giraffa*	*G. giraffa wardi*	LT628381, LT628408	[1]
SNNP	Zambia: Sioma Ngwezi NP	*G. giraffa giraffa*	*G. giraffa wardi*	LT628388, LT628415	[1]
LVNP8-01	Zambia: South Luangwa NP	*G. tippelskirchi thornicrofti*	*G. tippelskirchi thornicrofti*	HF571176, HF571134	[4]
LVNP8-02	Zambia: South Luangwa NP	*G. tippelskirchi thornicrofti*	*G. tippelskirchi thornicrofti*	HF571177, HF571135	[4]
LVNP8-03	Zambia: South Luangwa NP	*G. tippelskirchi thornicrofti*	*G. tippelskirchi thornicrofti*	HF571178, HF571136	[4]
LVNP8-04	Zambia: South Luangwa NP	*G. tippelskirchi thornicrofti*	*G. tippelskirchi thornicrofti*	HF571179, HF571137	[4]
LVNP8-05	Zambia: South Luangwa NP	*G. tippelskirchi thornicrofti*	*G. tippelskirchi thornicrofti*	HF571180, HF571138	[4]
LVNP8-06	Zambia: South Luangwa NP	*G. tippelskirchi thornicrofti*	*G. tippelskirchi thornicrofti*	HF571181, HF571139	[4]
LVNP8-08	Zambia: South Luangwa NP	*G. tippelskirchi thornicrofti*	*G. tippelskirchi thornicrofti*	HF571182, HF571140	[4]
LVNP8-09	Zambia: South Luangwa NP	*G. tippelskirchi thornicrofti*	*G. tippelskirchi thornicrofti*	HF571183, HF571141	[4]
LVNP8-13	Zambia: South Luangwa NP	*G. tippelskirchi thornicrofti*	*G. tippelskirchi thornicrofti*	HF571184, HF571142	[4]
LVNP8-14	Zambia: South Luangwa NP	*G. tippelskirchi thornicrofti*	*G. tippelskirchi thornicrofti*	HF571185, HF571143	[4]

Sample	Origin	Taxonomy [1]	Taxonomy [2]	Accession	Source
LVNP8-16	Zambia: South Luangwa NP	*G. tippelskirchi thornicrofti*	*G. tippelskirchi thornicrofti*	HF571186, HF571144	[4]
LVNP8-17	Zambia: South Luangwa NP	*G. tippelskirchi thornicrofti*	*G. tippelskirchi thornicrofti*	HF571187, HF571145	[4]
LVNP8-18	Zambia: South Luangwa NP	*G. tippelskirchi thornicrofti*	*G. tippelskirchi thornicrofti*	HF571188, HF571146	[4]
LVNP8-19	Zambia: South Luangwa NP	*G. tippelskirchi thornicrofti*	*G. tippelskirchi thornicrofti*	HF571189, HF571147	[4]
LVNP8-20	Zambia: South Luangwa NP	*G. tippelskirchi thornicrofti*	*G. tippelskirchi thornicrofti*	HF571190, HF571148	[4]
LVNP8-21	Zambia: South Luangwa NP	*G. tippelskirchi thornicrofti*	*G. tippelskirchi thornicrofti*	HF571191, HF571149	[4]
LVNP8-22	Zambia: South Luangwa NP	*G. tippelskirchi thornicrofti*	*G. tippelskirchi thornicrofti*	HF571192, HF571150	[4]
LVNP8-25	Zambia: South Luangwa NP	*G. tippelskirchi thornicrofti*	*G. tippelskirchi thornicrofti*	HF571193, HF571151	[4]
LVNP8-26	Zambia: South Luangwa NP	*G. tippelskirchi thornicrofti*	*G. tippelskirchi thornicrofti*	HF571194, HF571152	[4]
LVNP8-27	Zambia: South Luangwa NP	*G. tippelskirchi thornicrofti*	*G. tippelskirchi thornicrofti*	HF571195, HF571153	[4]
LVNP8-28	Zambia: South Luangwa NP	*G. tippelskirchi thornicrofti*	*G. tippelskirchi thornicrofti*	HF571196, HF571154	[4]
LVNP8-29	Zambia: South Luangwa NP	*G. tippelskirchi thornicrofti*	*G. tippelskirchi thornicrofti*	HF571197, HF571155	[4]
LVNP8-31	Zambia: South Luangwa NP	*G. tippelskirchi thornicrofti*	*G. tippelskirchi thornicrofti*	HF571198, HF571156	[4]
LVNP8-32	Zambia: South Luangwa NP	*G. tippelskirchi thornicrofti*	*G. tippelskirchi thornicrofti*	HF571199, HF571157	[4]
LVNP8-33	Zambia: South Luangwa NP	*G. tippelskirchi thornicrofti*	*G. tippelskirchi thornicrofti*	HF571200, HF571158	[4]
LVNP8-34	Zambia: South Luangwa NP	*G. tippelskirchi thornicrofti*	*G. tippelskirchi thornicrofti*	HF571201, HF571159	[4]
LVNP8-35	Zambia: South Luangwa NP	*G. tippelskirchi thornicrofti*	*G. tippelskirchi thornicrofti*	HF571202, HF571160	[4]
LVNP8-36	Zambia: South Luangwa NP	*G. tippelskirchi thornicrofti*	*G. tippelskirchi thornicrofti*	HF571203, HF571161	[4]
LVNP8-37	Zambia: South Luangwa NP	*G. tippelskirchi thornicrofti*	*G. tippelskirchi thornicrofti*	HF571204, HF571162	[4]
LVNP8-38	Zambia: South Luangwa NP	*G. tippelskirchi thornicrofti*	*G. tippelskirchi thornicrofti*	HF571205, HF571163	[4]
LVNP8-39	Zambia: South Luangwa NP	*G. tippelskirchi thornicrofti*	*G. tippelskirchi thornicrofti*	HF571206, HF571164	[4]
LVNP8-40	Zambia: South Luangwa NP	*G. tippelskirchi thornicrofti*	*G. tippelskirchi thornicrofti*	HF571207, HF571165	[4]
LVNP8-41	Zambia: South Luangwa NP	*G. tippelskirchi thornicrofti*	*G. tippelskirchi thornicrofti*	HF571208, HF571166	[4]
LVNP8-42	Zambia: South Luangwa NP	*G. tippelskirchi thornicrofti*	*G. tippelskirchi thornicrofti*	HF571209, HF571167	[4]
LVNP8-07	Zambia: South Luangwa NP	*G. tippelskirchi thornicrofti*	*G. tippelskirchi thornicrofti*	HM536643	Unpublished
LVNP8-15	Zambia: South Luangwa NP	*G. tippelskirchi thornicrofti*	*G. tippelskirchi thornicrofti*	HM536649	Unpublished
LVNP8-23	Zambia: South Luangwa NP	*G. tippelskirchi thornicrofti*	*G. tippelskirchi thornicrofti*	HM536655	Unpublished
LVNP8-24	Zambia: South Luangwa NP	*G. tippelskirchi thornicrofti*	*G. tippelskirchi thornicrofti*	HM536656	Unpublished
LVNP8-30	Zambia: South Luangwa NP	*G. tippelskirchi thornicrofti*	*G. tippelskirchi thornicrofti*	HM536662	Unpublished
LVNP8-10	Zambia: South Luangwa NP	*G. tippelskirchi thornicrofti*	*G. tippelskirchi thornicrofti*	MT605041	[10]
LVNP8-12	Zambia: South Luangwa NP	*G. tippelskirchi thornicrofti*	*G. tippelskirchi thornicrofti*	MT605042	[10]
SUN1	Zambia: Sun hotel, Livingstone	*G. giraffa giraffa*	*G. giraffa wardi*	LT628391, LT628418	[1]
SUN2	Zambia: Sun hotel, Livingstone	*G. giraffa giraffa*	*G. giraffa wardi*	LT628392, LT628419	[1]
SUN3	Zambia: Sun hotel, Livingstone	*G. giraffa giraffa*	*G. giraffa wardi*	LT628393, LT628420	[1]
SUN4	Zambia: Sun hotel, Livingstone	*G. giraffa giraffa*	*G. giraffa wardi*	LT628394, LT628421	[1]

**Sample**	**Origin**	**Taxonomy** [1]	**Taxonomy** [2]	**Accession**	**Source**
BVC001	Zimbabwe: Bubye Valley Conservancy	*G. giraffa angolensis*	*G. giraffa giraffa*	KY865101, KY865138	[9]
BVC002	Zimbabwe: Bubye Valley Conservancy	*G. giraffa angolensis*	*G. giraffa giraffa*	KY865102, KY865139	[9]
BVC003	Zimbabwe: Bubye Valley Conservancy	*G. giraffa angolensis*	*G. giraffa giraffa*	KY865103, KY865140	[9]
BVC004	Zimbabwe: Bubye Valley Conservancy	*G. giraffa angolensis*	*G. giraffa giraffa*	KY865104, KY865141	[9]
BVC005	Zimbabwe: Bubye Valley Conservancy	*G. giraffa angolensis*	*G. giraffa giraffa*	KY865105, KY865142	[9]
BVC006	Zimbabwe: Bubye Valley Conservancy	*G. giraffa angolensis*	*G. giraffa giraffa*	KY865106, KY865143	[9]
BVC007	Zimbabwe: Bubye Valley Conservancy	*G. giraffa angolensis*	*G. giraffa giraffa*	KY865107, KY865144	[9]
BVC008	Zimbabwe: Bubye Valley Conservancy	*G. giraffa angolensis*	*G. giraffa giraffa*	KY865108, KY865145	[9]
BVC009	Zimbabwe: Bubye Valley Conservancy	*G. giraffa angolensis*	*G. giraffa giraffa*	KY865109, KY865146	[9]
BVC010	Zimbabwe: Bubye Valley Conservancy	*G. giraffa angolensis*	*G. giraffa giraffa*	KY865110, KY865147	[9]
Okapi	NA	*Okapia johnstoni*	*Okapia johnstoni*	JN632674	[5]

Table S2. Sample details and mapping statistics for re-sequenced individuals. Sample identifier, sampling location, taxonomic classification, accession number, original data source, total number of reads, percentage of mapped reads, percentage of duplicated reads, median insert size, and depth of coverage (before and after cleaning the BAM files) are shown per individual.

Sample	Origin	Taxonomy [1]	Accession	Source	Total reads (10^6)	Mapped (%)	Dup. (%)	Ins. size (bp)	Depth (×)	
									Raw	Clean
Niger02	Kouré, Niger	*Giraffa camelopardalis peralta*	SRR16477187	This study	301.8	99.2	21.5	353	13	12
Niger03	Kouré, Niger	*Giraffa camelopardalis peralta*	SRR16477186	This study	301.7	99.2	19.7	406	14	13
Niger06	Kouré, Niger	*Giraffa camelopardalis peralta*	SRR16477185	This study	311.3	99.3	20.3	367	14	13
Niger07	Kouré, Niger	*Giraffa camelopardalis peralta*	SRR16477184	This study	283.7	99.2	22.8	362	12	11
Niger12	Kouré, Niger	*Giraffa camelopardalis peralta*	SRR16477183	This study	283.8	99.3	22.9	347	12	11
WA720	Kouré, Niger	*Giraffa camelopardalis peralta*	SRR11905169	[10]	419.8	98.1	23.4	277	18	15
WA733	Kouré, Niger	*Giraffa camelopardalis peralta*	SRR11905168	[10]	317.6	97.2	16.2	312	15	13
WA746	Kouré, Niger	*Giraffa camelopardalis peralta*	SRR11905157	[10]	275.1	97.9	13.6	284	13	11
WA806	Kouré, Niger	*Giraffa camelopardalis peralta*	SRR11905146	[10]	397.9	99.1	22.0	282	17	15
WA808	Kouré, Niger	*Giraffa camelopardalis peralta*	SRR11905135	[10]	347.9	98.6	15.3	304	16	15
GNP01	Garamba NP, DR Congo	*Giraffa camelopardalis antiquorum*	SRR11905126	[10]	340.5	99.2	16.8	303	16	14
GNP04	Garamba NP, DR Congo	*Giraffa camelopardalis antiquorum*	SRR11905125	[10]	378.1	99.0	16.5	316	18	16
GNP05	Garamba NP, DR Congo	*Giraffa camelopardalis antiquorum*	SRR11905124	[10]	419.3	99.2	16.6	291	19	17
SNR2	Shambe NP, South Sudan	*Giraffa camelopardalis antiquorum*	SRR11905123	[10]	353.6	99.0	17.2	304	16	14
ZAK01	Zakouma NP, Chad	*Giraffa camelopardalis antiquorum*	SRR16477182	This study	307.7	99.2	20.2	383	14	13
ZAK02	Zakouma NP, Chad	*Giraffa camelopardalis antiquorum*	SRR16477181	This study	279.3	99.1	23.5	378	12	11
ZAK03	Zakouma NP, Chad	*Giraffa camelopardalis antiquorum*	SRR16477180	This study	304.6	99.2	20.6	370	14	12
ZAK04	Zakouma NP, Chad	*Giraffa camelopardalis antiquorum*	SRR16477179	This study	313.1	99.2	21.5	354	14	12
ZAK07	Zakouma NP, Chad	*Giraffa camelopardalis antiquorum*	SRR16477178	This study	308.7	99.2	20.7	353	14	12
ZNP01	Zakouma NP, Chad	*Giraffa camelopardalis antiquorum*	ERR1248124	[1]	547.5	98.7	3.4	474	26	23
ETH1	Gambella NP, Ethiopia	*Giraffa camelopardalis camelopardalis*	SRR11905122	[10]	400.9	99.3	15.0	289	19	17
ETH2	Gambella NP, Ethiopia	*Giraffa camelopardalis camelopardalis*	SRR11905167	[10]	346.3	99.0	17.2	300	16	14
ETH3	Gambella NP, Ethiopia	*Giraffa camelopardalis camelopardalis*	SRR11905166	[10]	350.1	99.1	16.4	300	16	15
MF06	Murchison Falls NP, Uganda	*Giraffa camelopardalis camelopardalis*	SRR11905165	[10]	329.4	98.3	14.3	283	16	14
MF22	Murchison Falls NP, Uganda	*Giraffa camelopardalis camelopardalis*	SRR11905164	[10]	537.1	99.1	19.6	319	24	22
MF24	Murchison Falls NP, Uganda	*Giraffa camelopardalis camelopardalis*	SRR11905163	[10]	498.0	99.1	10.9	320	25	23
WOAK	White Oak Conservation Center	*Okapia johnstoni*	SRR3217625, SRR3217884	[12]	437.8	91.5	5.3	534	21	19

References

1. Fennessy, J.; Bidon, T.; Reuss, F.; Kumar, V.; Elkan, P.; Nilsson, M.A.; Vamberger, M.; Fritz, U.; Janke, A. Multi-locus analyses reveal four giraffe species instead of one. *Curr. Biol.* **2016**, *26*, 2543–2549, doi:10.1016/j.cub.2016.07.036.
2. Petzold, A.; Magnant, A.-S.; Edderai, D.; Chardonnet, B.; Rigoulet, J.; Saint-Jalme, M.; Hassanin, A. First insights into past biodiversity of giraffes based on mitochondrial sequences from museum specimens. *Eur. J. Taxon.* **2020**, doi:10.5852/ejt.2020.703.
3. Bock, F.; Fennessy, J.; Bidon, T.; Tutchings, A.; Marais, A.; Deacon, F.; Janke, A. Mitochondrial sequences reveal a clear separation between Angolan and South African giraffe along a cryptic rift valley. *BMC Evol. Biol.* **2014**, *14*, 219, doi:10.1186/s12862-014-0219-7.
4. Fennessy, J.; Bock, F.; Tutchings, A.; Brenneman, R.; Janke, A. Mitochondrial DNA analyses show that Zambia's South Luangwa Valley giraffe (*Giraffa camelopardalis thornicrofti*) are genetically isolated. *Afr. J. Ecol.* **2013**, *51*, 635–640, doi:10.1111/aje.12085.
5. Hassanin, A.; Delsuc, F.; Ropiquet, A.; Hammer, C.; Jansen van Vuuren, B.; Matthee, C.; Ruiz-Garcia, M.; Catzeflis, F.; Areskoug, V.; Nguyen, T.T.; et al. Pattern and timing of diversification of Cetartiodactyla (Mammalia, Laurasiatheria), as revealed by a comprehensive analysis of mitochondrial genomes. *C. R. Biol.* **2012**, *335*, 32–50, doi:10.1016/j.crvi.2011.11.002.
6. Hassanin, A.; Ropiquet, A.; Gourmand, A.-L.; Chardonnet, B.; Rigoulet, J. Mitochondrial DNA variability in *Giraffa camelopardalis*: consequences for taxonomy, phylogeography and conservation of giraffes in West and Central Africa. *C. R. Biol.* **2007**, *330*, 265–274, doi:10.1016/j.crvi.2007.02.008.
7. Winter, S.; Fennessy, J.; Janke, A. Limited introgression supports division of giraffe into four species. *Ecol. Evol.* **2018**, *8*, 10156–10165, doi:10.1002/ece3.4490.
8. Brown, D.M.; Brenneman, R.A.; Koepfli, K.-P.; Pollinger, J.P.; Milá, B.; Georgiadis, N.J.; Louis, E.E.; Grether, G.F.; Jacobs, D.K.; Wayne, R.K. Extensive population genetic structure in the giraffe. *BMC Biol.* **2007**, *5*, 57, doi:10.1186/1741-7007-5-57.
9. Winter, S.; Fennessy, J.; Fennessy, S.; Janke, A. Matrilineal population structure and distribution of the Angolan giraffe in the Namib desert and beyond. *Ecol. Genet. Genomics* **2018**, *7–8*, 1–5, doi:10.1016/j.egg.2018.03.003.
10. Coimbra, R.T.F.; Winter, S.; Kumar, V.; Koepfli, K.-P.; Gooley, R.M.; Dobrynin, P.; Fennessy, J.; Janke, A. Whole-genome analysis of giraffe supports four distinct species. *Curr. Biol.* **2021**, *31*, 2929-2938.e5, doi:10.1016/j.cub.2021.04.033.
11. Winter, S.; Coimbra, R.T.F.; Bronec, A.; Hay, C.; Salb, A.L.; Fennessy, J.; Janke, A. Species assignment and conservation genetics of giraffe in the Republic of Malawi. *Conserv. Genet.* **2019**, *20*, 665–670, doi:10.1007/s10592-018-01142-4.
12. Agaba, M.; Ishengoma, E.; Miller, W.C.; McGrath, B.C.; Hudson, C.N.; Bedoya Reina, O.C.; Ratan, A.; Burhans, R.; Chikhi, R.; Medvedev, P.; et al. Giraffe genome sequence reveals clues to its unique morphology and physiology. *Nat. Commun.* **2016**, *7*, 11519, doi:10.1038/ncomms11519.
